# Erratic Maternal Care Induces Avoidant-Like Attachment Deficits in a Mouse Model of Early Life Adversity

**DOI:** 10.1523/ENEURO.0249-25.2025

**Published:** 2025-11-06

**Authors:** Zoë A. MacDowell Kaswan, Christian Bowers, Ivan Teplyakov, Ayna Sibtain, Jose Munoz-Martin, Sahabuddin Ahmed, Lily Kaffman, Lauryn Giuliano, Marcelo O. Dietrich, Arie Kaffman

**Affiliations:** ^1^Department of Psychiatry, Yale University School of Medicine, New Haven, Connecticut 06511; ^2^Department of Cellular Biology, Genetics, and Physiology, University of Malaga, Malaga 29010, Spain; ^3^The Biomedical Research Institute of Malaga and Nanomedicine Platform (IBIMA BIONAND Platform), Malaga 29590, Spain; ^4^Laboratory of Physiology of Behavior, Department of Comparative Medicine, School of Medicine, Yale University, New Haven, Connecticut 06520; ^5^Departments of Comparative Medicine, Yale University, New Haven Connecticut 06520; ^6^Neuroscience, School of Medicine, Yale University, New Haven Connecticut 06520; ^7^Interdepartmental Neuroscience Program, Yale University School of Medicine, New Haven Connecticut 06520

**Keywords:** attachment, corticosterone, early life adversity, limited bedding and nesting, mice

## Abstract

Attachment theory offers an important clinical framework for understanding and treating negative effects of early life adversity. Attachment styles emerge during critical periods of development in response to caregivers' ability to consistently meet their offspring’s needs. Attachment styles are classified as secure or insecure (anxious, avoidant, or disorganized), with rates of insecure attachment rising in high-risk populations and correlating with a plethora of negative health outcomes throughout life. Despite its importance, little is known about the neural basis of attachment. Work in rats has demonstrated that limited bedding and nesting (LB) impairs maternal care and produces abnormal maternal attachment linked to increased pup corticosterone. However, the effects of LB on attachment-like behavior have not been investigated in mice where additional genetic and molecular tools are available. Furthermore, no group has utilized home-cage monitoring to link abnormal maternal care with deficits in attachment-like behavior. Using home-cage monitoring, we confirmed a robust increase in maternal fragmentation among LB dams. Abnormal maternal care was correlated with elevated corticosterone levels on postnatal day 7 (P7) and a stunted growth trajectory that persisted later in life. LB did not alter maternal buffering at P8 or maternal preference at P18, indicating that certain attachment-like behaviors remain unaffected despite exposure to high levels of erratic maternal care. However, LB male and female pups vocalized less in response to maternal separation at P8, did not readily approach their dam at P13, and exhibited higher anxiety-like behavior at P18, suggesting that LB induces avoidant-like attachment deficits in mice.

## Significance Statement

The impoverished conditions of limited bedding and nesting (LB) cause erratic maternal care and elevated corticosterone levels in rat and mouse pups. The increase in corticosterone levels causes attachment-like deficits in rat pups; however, it remains unclear whether similar deficits are observed in mice, where additional genomic and molecular tools are available. Using continuous home-cage monitoring, we confirmed a substantial increase in erratic maternal care and elevated corticosterone levels in 7-day-old mouse pups. LB mouse pups exhibited attachment-like deficits in some, but not all, tests, underscoring the robustness of this evolutionarily conserved bond. Despite some similarities, the attachment abnormalities observed in mice differed from previous reports in rats, paving the way for in-depth mechanistic studies in mice.

## Introduction

According to attachment theory, reciprocal interactions between a caregiver and their offspring during a critical developmental period establish a strong emotional bond that is essential for survival and normal development (Bowlby, 1978; Izaki et al., 2024). A typical attachment sequence begins with distress vocalizations from the offspring in response to homeostatic perturbations, leading to proximity-seeking behaviors that bring the offspring and the caregiver closer together to alleviate distress. The repeated successful completion of this sequence creates a secure base from which the offspring can explore their environment (Hornor, 2019; Sullivan and Opendak, 2021; Izaki et al., 2024).

Using the Strange Situation paradigm as a standardized procedure for observing infant responses to separation and reunification from the caregiver, Ainsworth and colleagues identified four attachment styles: secure, anxious, avoidant, and disorganized, with anxious, avoidant, and disorganized styles sometimes grouped together as insecure attachment (Hornor, 2019; Gregory et al., 2020). Secure attachment occurs when caregivers consistently meet the child’s physical and emotional needs, which is manifested by the caregiver’s capacity to effectively console and reduce distress upon reunification. Anxiously attached children exhibit excessive distress when the caregiver leaves and are difficult to console upon reunion. In contrast, avoidantly attached children display minimal distress when the caregiver departs and may avoid or even express anger toward the caregiver during reunion. Children with disorganized attachment demonstrate a combination of insecure and avoidant behaviors (Hornor, 2019; Gregory et al., 2020). Avoidant attachment is frequently associated with severe childhood neglect (Mikulincer and Shaver, 2016; Widom et al., 2018; Midolo et al., 2020; Tian et al., 2025). Moreover, rates of disorganized attachment can reach as high as 35% among children who have experienced severe neglect or maltreatment (Wright and Edginton, 2016), and this pattern is strongly correlated with increased risk for psychiatric disorders and somatization later in life (Hornor, 2019; Gregory et al., 2020).

Attachment-based randomized controlled trials have been shown to increase rates of secure attachment and to improve psychiatric comorbidities in high-risk populations (Wright and Edginton, 2016; Diamond et al., 2019; Gregory et al., 2020; Hepworth et al., 2020). Additionally, these trials have been effective in improving medical comorbidities (Hornor, 2019; Gregory et al., 2020), including reducing inflammation (Ross et al., 2021; Londono Tobon et al., 2023) and decreasing stunting (Noviana et al., 2023), further demonstrating the clinical utility of this approach.

Despite the significance of attachment formation during development, our understanding of its underlying biology remains limited. This includes the impact of attachment on brain circuits that regulate mood and cognition, as well as more general processes such as immune function and growth (Izaki et al., 2024). The evolutionarily conserved nature of attachment suggests that rodent models can offer valuable insights into these questions (Sullivan and Opendak, 2021). Studies in rats have shown that erratic maternal care induced by limited bedding and nesting material (LB) leads to elevated pup corticosterone levels that are directly responsible for abnormal amygdala activation and deficits in proximity-seeking behavior (Raineki et al., 2019). However, to the best of our knowledge, the effects of LB on maternal attachment have not yet been investigated in mice, where additional genetic tools could be utilized to study the neurobiology of attachment behavior.

The goals of the present study were twofold: first, to establish a reproducible and unbiased method for quantifying maternal behavior and second, to develop a longitudinal series of tests to assess maternal attachment in mouse pups using a modified LB paradigm (Dayananda et al., 2022; Ahmed et al., 2024; Islam et al., 2024; Ahmed et al., 2025). This modified paradigm extends the period of exposure to impoverished conditions from birth (postnatal day 0, P0) through P25, producing structural and functional alterations that parallel those observed in children exposed to neglect (Dayananda et al., 2022; Ahmed et al., 2024; Islam et al., 2024; Ahmed et al., 2025). We found that maternal movement and fragmentation during the first week of life were highly quantifiable and reproducible using a home-cage monitoring system and present data suggesting that LB pups display avoidant-like attachment deficits. Together, this study provides a foundation to further investigate the mechanisms by which erratic maternal care disrupts attachment behavior in the mouse.

## Materials and Methods

### Animals

C57BL/6J (Jackson Laboratories, stock #000664) mice were housed in standard Plexiglas cages and kept on a standard 12 h light–dark cycle (lights on at 07:00 A.M.) with constant temperature 20 ± 1°C and humidity 43% ± 2, with food and water provided *ad libitum*.

All studies were approved by the Institutional Animal Care and Use Committee (IACUC) at Yale University and were conducted in accordance with the recommendations of the NIH Guide for the Care and Use of Laboratory Animals.

### Limited bedding

C57BL/6J mice were mated using a 3:1 female-to-male ratio in standard mouse Plexiglas cages layered with 500 cc of corncob bedding and a nestlet. Visibly pregnant females were placed individually in Noldus PhenoTyper cages for mice (Noldus Technology) ∼2–4 d before birth and given 500 cc softcob bedding (catalog #7087C, Inotiv Teklad), a nestlet, and 23 chow pellets on the floor of the cage. The PhenoTypers are a home-cage monitoring system composed of a 30 × 30 cm cage with side-mounted food and water, and a lid with built-in infrared lights and an infrared camera. The PhenoTypers are directly connected to Noldus EthoVision XT 17 (Noldus Technology) software for automated continuous mouse tracking. The nest region of each arena was assigned in EthoVision and beginning at birth, designated as P0, the dams' total movement, time in the nest, and frequency of entrances/exists to the nest were recorded continuously until P2 (preassignment period, black rectangle in Fig. 1*A*). At P2, litters were culled to 5–8 pups per litter and randomly assigned to either control (CTL, black color in figures) or LB (red color in figures) conditions. CTL litters received 500 cc of softcob bedding, a nestlet, and 15 cc soiled bedding from the old cage. LB litters received 125 cc softcob bedding, no nestlet, and 15 cc soiled bedding from the old cage. Softcob bedding (corncob bedding supplemented with small pieces of nesting material) was used in the PhenoTypers, as pilot experiments indicated that LB litters housed on corncob bedding alone exhibited high pup mortality. Dam behavior was recorded using EthoVision until P7 (postassignment period, black and red rectangles in all timelines). At P7, litters were transferred to standard mouse Plexiglas cages (black and red lines in all timelines), while maintaining CTL and LB conditions using normal corncob bedding. CTL litters received 500 cc of corncob bedding and a nestlet, whereas LB litters received 125 cc corncob bedding without nestlet as previously described (Ahmed et al., 2024; Islam et al., 2024). CTL and LB litters raised under standard caging conditions were not placed in the PhenoTyper at P2 but instead remained in standard Plexiglas cages and were randomized to CTL (500 cc of corncob bedding and a nestlet) or LB conditions (125 cc of corncob bedding without a nestlet). Bedding was changed at P14 and P21. CTL and LB assignments were terminated at weaning. Litters were weaned at P26 and housed with 2–5 same-sex littermates in standard Plexiglass mouse cages under CTL conditions (i.e., 500 cc of corncob bedding and a nestlet), with 2–3 pellets of chow placed on the floor of the cage.

### Quantification of maternal behavior

Separate detection settings were required for light and dark cycles. For Cohort 1 (used for longitudinal behavior), videos of dam behavior were analyzed after recording. An observer watched EthoVision’s automated tracking and, when necessary, adjusted the detection settings for accuracy and moved the assigned nest zone if the dam moved her nest. For Cohorts 2 and 3 (used for corticosterone and maternal affiliation, respectively), automated mouse tracking was performed in real time and detection settings were automatically switched between the room’s light and dark cycles. After data collection, videos were checked to ensure that the dam kept her nest in the pre-assigned area. If the dam moved her nest, the recording time was noted, assigned nest zone was moved in EthoVision, and the data were reanalyzed. Dams' mean movement, cumulative times in Arena and nest zones, and frequency in the nest zone were automatically calculated by EthoVision in 1 h time bins. If the last time bin was incomplete, it was discarded. In some instances, EthoVision failed to detect the dam when she was immobile on the nest for long periods of time. Therefore, any time that the dam was not detected in the arena, she was assumed to be on the nest.

To assess the accuracy of automated scoring, a subset of 12 litters (*N* = 6 CTL, 6 LB; three litters per condition for each of Cohorts 2 and 3) were hand-scored for 1 h each. In addition to quantifying times that the dam entered/exited the nest and time on the nest, EthoVision’s tracking was checked using the “Integrated Visualization” view. Hand-scoring results were compared with the automated results during the same hour. Hours for analysis were chosen at random, but covered P2–P6, and six different recording hours during the dark phase. The same set of six day/time pairings were used for both CTL and LB. The dark phase was chosen for this analysis because, during scoring for Cohort 1, errors in tracking were observed to be more common during the dark phase.

### Blood collection and corticosterone ELISA (P7)

Blood was collected at 14:00–15:00 of P7 pups using rapid decapitation. To minimize stress, blood was collected in a separate room. Blood was first collected from the dam, followed by pups, which were individually transferred in a cup containing home-cage bedding. After allowing the blood to clot for 30 min at room temperature, samples were centrifuged for 5 min at 2,400 × *g* at room temperature. Serum was collected and stored at −80°C until use. At least one male and one female pup per litter, of approximately average weight for that litter, for a total of 7–8 pups per sex per rearing condition were selected for corticosterone analysis. Serum corticosterone levels were measured in duplicates using ELISA kit (catalog # K014-H1, Arbor Assays), according to the manufacturer’s instructions, using the 100 μl format.

### Buffering of ultrasonic vocalizations (P8)

The extent to which a female’s presence buffered pups' ultrasonic vocalizations was tested at P8 (i.e., one day after litters were transferred to standard Plexiglas cages; Fig. 4*A*). Testing was done between 13:00 and 17:00 under standard room illumination (600 lux). During the isolation phase, pups were placed individually in an empty soundproofed standard Plexiglass cage and ultrasonic vocalizations (USVs) were recorded for 5 min using UltraSoundGate condenser microphone CM 16 connected via an UltraSoundGate 416 USGH audio device (Avisoft Bioacoustics). After 5 min of isolation, an anesthetized unrelated, but experienced, adult female (“aunt”), fed the same diet as the dam, was placed on her belly in physical contact with the pup for 5 min, and USVs were recorded for an additional 5 min (Fig. 4*B*, “With Aunt” phase). Aunts were anesthetized using 10/0.1 mg/kg ketamine/xylazine mixture dissolved in water. At the completion of the recordings, pups were weighed and returned to the home and isolation chambers cleaned with 70% ethanol in between pups. Numbers of USVs were quantified using VocalMat software (Fonseca et al., 2021) and “buffering” was calculated using the following formula: % Buffering = (# USVs with aunt − # USVs in isolation) / # USVs in isolation × 100.

### Maternal affiliation (P13)

At P13, a separate cohort of pups (Cohort 3) were tested for proximity-seeking behavior to their anesthetized biological dam, termed “maternal affiliation.” All pups were killed after the completion of the maternal affiliation test. Testing was done between 13:00 and 18:00 under standard room illumination (600 lux). Dams were anesthetized with urethane (1.5 mg/kg) and placed in a standardized Plexiglass mouse cage layered with 100 cc clean corncob bedding mixed with 30 cc of dirty home-cage bedding. The addition of dirty bedding was necessary to prevent most pups from freezing. Pups were then individually placed at one end of the arena and allowed 5 min to freely explore the cage. Pup behavior was recorded using an overhead HD Pro Webcam C920 (Logitech), and videos were analyzed using EthoVision XT 17 (Noldus Technology) to determine the total time spent at the dam’s ventral or dorsal side (summed to calculate total interaction time) latency to approach and mean distance between pup and dam (calculated as mean distance between pup and closer of either the dorsal or ventral zone). For binary approach versus no approach analysis, a pup was considered to have approached if it entered either the dorsal or ventral zones.

### Open field and maternal preference tests (P18)

P18 pups were tested in the open field and maternal preference tests between 13:00 and 17:00 under standard room illumination (∼600 lux). In the first phase, the pup was given 5 min to explore the empty arena (20 × 42 cm). This served both as an open field test and habituation to the arena. The pup was then weighed, individually marked, and returned to the home cage. Immediately following this habituation, the dam and a novel object were placed under wire pencil cups at opposite ends of the arena. The pup was placed in the center of the arena and given 5 min to freely investigate (Fig. 6*B*). Location of the dam and novel object were switched between litters to reduce bias. Both phases of the test were recorded using an overhead HD Pro Webcam C920 (Logitech) and analyzed using EthoVision XT 17 (Noldus Technology). For the open field test, total movement and percent time in center (a central region 1/2 the width and length of the total arena) were calculated. For the maternal preference test, total movement and the amount of time the pup spent in ∼2 cm radius (“zone”) around either the pencil cup containing an awake dam or novel object was quantified. Note that dams used in the maternal preference test were never anesthetized. Preference index was calculated as follows: (time spent in dam’s zone) × 100 / (time spent in dam’s zone + time spent in novel object’s zone). Thus, preference index >50% indicates preference for the dam.

### Social interaction (P33–34)

The social interaction test was performed in adolescent mice ages P33-34. Testing was performed between 13:00 and 17:00 under standard room illumination (600 lux). Experimental animals (e.g., CTL or LB) were allowed 10 min to explore the empty arena (20 × 42 cm). After 10 min of habituation, an age- and sex-matched stranger mouse was added to the arena (Fig. 7*B*), and social exploration was recorded for 10 min using an overhead HD Pro Webcam C920 (Logitech). Latency to first interaction initiated by the experimental mouse and total time that the experimental mouse interacted with the stranger were quantified by an observed blind to rearing condition or sex.

### Experimental design and statistical analysis

Data were graphed and statistical analyses were performed using GraphPad Prism 10 (GraphPad Software). Maternal behavior was analyzed using a 2 × 2 repeated-measures (rm) ANOVA, with rearing as a between-subjects variable and assignment as a within-subjects variable (Fig. 1*D*,*F*,*H*). Significant rearing by assignment interactions were followed by Fisher’s least significant difference (LSD) post hoc analyses. A three-way rmANOVA was employed to assess the effects of rearing (between-subjects variable), day (within-subjects variable), and light–dark phase (within-subjects variable) on maternal behavior, with significant interactions followed by Tukey’s HSD post hoc analyses (Fig. 1*E*,*G*,*I*). Three-way rmANOVAs were also utilized to determine the effects of rearing, sex, and test phase (Fig. 4*C*) or side preference (Fig. 5*G*). Two-way ANOVAs were conducted to examine the effects of rearing and sex on weight at different ages (Fig. 2*A*), corticosterone levels (Fig. 3*B*), maternal buffering (Fig. 4*D*), maternal affiliation (Fig. 5*D–F*), exploratory behavior in the open field (Fig. 6*C*,*D*), maternal preference index (Fig. 6*E*,*F*), and social interaction (Fig. 7*B*,*C*). Pearson’s correlations were calculated to characterize the relationship between the dams’ behavior and the following variables: body weight (Fig. 2*B–F*), corticosterone levels at P8, maternal affiliation at P13, time spent in the center of the open field, and maternal preference at P18. Chi-square analysis was used to assess the effect of rearing on the approach versus no approach analysis in the maternal affiliation test (Fig. 5*C*).

## Results

### LB increased distance traveled and maternal fragmentation

Total distance traveled, maternal fragmentation, and time in nest were calculated for each condition during the pre- and postassignment periods across three cohorts (Fig. 1*A*; *n* = 20–21 litters/condition). Cohort 1 (*n* = 6 litters/condition) was used for longitudinal behavior (P8, P18, and P33), Cohort 2 (*n* = 9 litters/condition) was used for P7 corticosterone measurements, and Cohort 3 (*n* = 6 CTL, 5 LB litters) was used for P13 behavior. Consistent with prior reports (Bath et al., 2016; Walker et al., 2017; Gallo et al., 2019; Demaestri et al., 2022), LB dams exhibited a high degree of maternal fragmentation, characterized by frequent transitions in and out of the nest (Fig. 1*B*; Movies 1 and 2). LB dams gathered all available bedding and nesting material to form a rudimentary nest; however, pups were often found scattered across the cage due to being dragged outside the nest while suckling (Fig. 1*C*, Movie 3).

**Figure 1.**
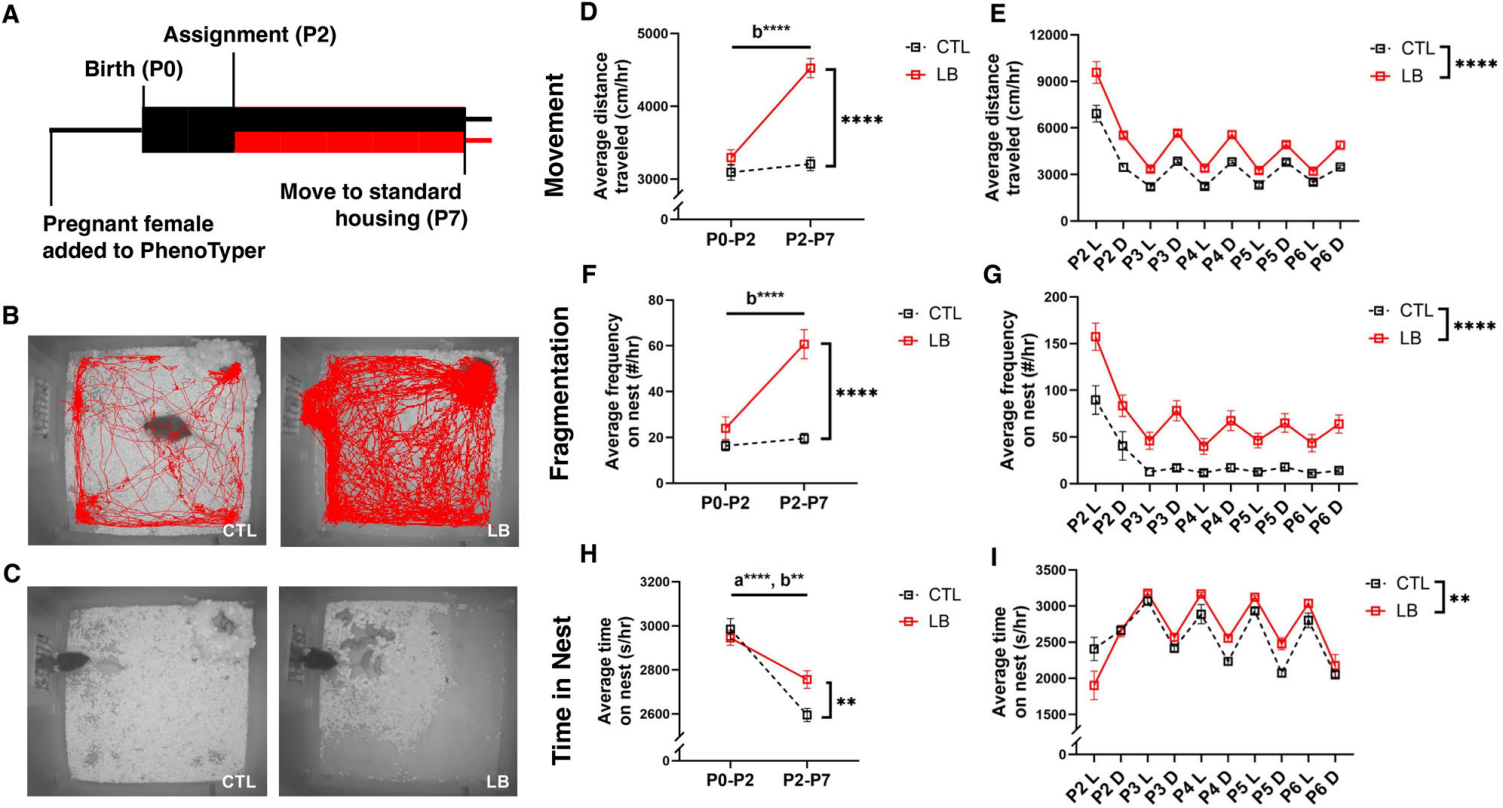
LB increases distance traveled, maternal fragmentation, and time in nest. ***A***, PhenoTyper timeline. Boxes indicate recording periods, and lines indicate nonrecording periods. Black denotes CTL conditions, and red denotes LB conditions. ***B***, Representative tracing of the dam’s movement from the 2nd to 3rd hour of the dark phase of P3. ***C***, Representative images of dams and their litters in the PhenoTypers during the dark phase of P3. ***D***, Average hourly distance traveled by the dam pre- (P0–P2) and postassignment (P2–P7). Rearing × assignment interaction: *F*_(1,18)_ = 35.42, *p* < 0.0001. Post hoc analysis: CTL versus LB (P0–P2): *p* = 0.14; CTL versus LB (P2–P7): *p* < 0.0001; CTL (P0–P2) versus (P2–P7): *p* = 0.39; LB (P0–P2 vs P2–P7): *p* < 0.0001. ***E***, Average hourly distance traveled by the dam during the light (L) and dark (D) phases after condition assignment. Day: *F*_(1.617,124.9)_ = 125.7, *p* < 0.0001; Rearing: *F*_(1,78)_ = 88.59, *p* < 0.0001; Light–dark cycle: *F*_(1,78)_ = 14.98, *p* = 0.0002; day × light–dark interaction: *F*_(4,309)_ = 126.1, *p* < 0.0001; rearing × light–dark: nonsignificant (NS); day × light–dark × rearing interaction: NS. Post hoc analysis: P2 versus all other days: *p* < 0.0001. ***F***, Average hourly maternal fragmentation pre- (P0–P2) and postassignment (P2–P7). Rearing × assignment interaction: *F*_(1,18)_ = 26.72, *p* < 0.0001. Post hoc analysis: CTL versus LB (P0–P2): *p* = 0.16; CTL versus LB (P2–P7): *p* < 0.0001; CTL (P0–P2) versus (P2–P7): *p* = 0.49; LB (P0–P2 vs P2–P7): *p* < 0.0001. ***G***, Average hourly maternal fragmentation during the light (L) and dark (D) phases after condition assignment. Day: *F*_(1.727,133.4)_ = 44.55, *p* < 0.0001; rearing: *F*_(1,78)_ = 69.06, *p* < 0.0001; light–dark cycle: NS; day × rearing interaction: NS; rearing × light–dark: *F*_(4,309)_ = 19.45, *p* < 0.0001; day × light–dark × rearing interaction: NS. Post hoc analysis: P2 versus all other days: *p* < 0.0001. ***H***, Average hourly time on nest pre- (P0–2) and postassignment (P2–7). Rearing assignment interaction: F_(1,38)_ = 6.84, *p* = 0.013. Post hoc analysis: CTL versus LB (P0–P2): *p* = 0.48; CTL versus LB (P2–P7): *p* = 0.0040; CTL (P0–P2) versus (P2–P7): *p* < 0.0001; LB (P0–P2 vs P2–P7): *p* = 0.0015. ***I***, Average hourly time on nest during the light (L) and dark (D) phases after condition assignment. Day: *F*_(2.596,199.9)_ = 14.78, *p* < 0.0001; rearing: *F*_(1,78)_ = 8.08, *p* = 0.0057, light–dark cycle: *F*_(1,78)_ = 102.6, *p* < 0.0001, day × rearing: *F*_(4,308)_ = 7.59, *p* < 0.0001; day × light–dark: *F*_(4,308)_ = 43.42, *p* < 0.0001; rearing × light–dark: NS; day × light–dark × rearing interaction: NS. *N* = 19–21 per rearing condition, 2 × 2 rmANOVA for ***D***, ***F***, ***H*** with a and b representing effects of pre- and postassignments for CTL and LB, respectively. Three-way rmANOVA for ***E***, ***G***, and ***I***. NS, nonsignificant, *p* > 0.05. See Extended Data Figures 1-1–1-4 for more details.

10.1523/ENEURO.0249-25.2025.f1-1 Figure 1-1 **Differences in Maternal Behavior Are More Prominent During the Dark Phase.** Maternal behavior is shown for the light and dark phases of P3-6. **(A)** Average hourly distance travelled. Rearing: F(1, 39) = 62.72, P < 0.0001; Light/dark: F(1, 39) = 266.8, P < 0.0001; Subject: F(39, 39) = 2.28, P = 0.0058; Rearing x Light/dark interaction: F(1, 39) = 7.96, P = 0.0075. Post-hoc analysis: CTL vs LB (light phase), CTL vs LB (dark phase), Light vs Dark (CTL), and Light vs Dark (LB) all P < 0.0001. **(B)** Average hourly frequency on the nest. Rearing: F(1, 39) = 34.28, P < 0.0001; Light/dark: F(1, 39) = 28.61, P < 0.0001; Subject: F(39, 39) = 7.07, P < 0.0001; Rearing x Light/dark interaction: F(1, 39) = 14.14, P = 0.0006. Post-hoc analysis: CTL vs LB (light phase) P < 0.0001, CTL vs LB (dark phase) P < 0.0001, Light vs Dark (CTL) P = 0.26, Light vs Dark (LB) P < 0.0001. **(C)** Average hourly time on the nest. Rearing: F(1, 39) = 14.66, P = 0.0005; Light/dark: F(1, 39) = 446.2, P < 0.0001; Subject: F(39, 39) = 2.63, P = 0.0016; Rearing x Light/dark interaction: F(1, 39) = 5.22, P = 0.028. Post-hoc analysis: CTL vs LB (light phase) P = 0.43, CTL vs LB (dark phase) P < 0.0001, Light vs Dark (CTL) P < 0.0001, Light vs Dark (LB) P < 0.0001. N = 21 litters per rearing condition. Analyzed by 2 × 2 rmANOVA with post-hoc uncorrected Fisher’s least significant difference test. Download Figure 1-1, TIF file.

10.1523/ENEURO.0249-25.2025.f1-2 Figure 1-2 **Reproducible and Robust Effects of Rearing Observed for Distance Traveled and Maternal Fragmentation, But Not for Time on Nest**. Effects on maternal movement (**A**), fragmentation (**B**), and time on nest (**C**) for the three cohorts tested. Cohort 1 (n = 6 litters/condition) was used for longitudinal behavior (P8, P18, & P33), cohort 2 (n = 9 litters/condition) was used for P7 corticosterone measurements, and cohort 3 (n = 6 CTL, 5 LB litters) was used for P13 maternal affiliation behavior. Download Figure 1-2, TIF file.

10.1523/ENEURO.0249-1125.2025.f1-3 Figure 1-3 **Effects of Rearing on Variance in Dam’s Behavior from P2 to P7. (A)** Variance in movement, F (19,20) = 2.20, P = 0.13. **(B)** Variance in time on nest, F (19,20) = 1.69, P = 0.25. (**C**) Variance in fragmentation, F (19,20) = 7.47, P < 0.0001. N = 20-21 litters per rearing condition. Download Figure 1-3, TIF file.

10.1523/ENEURO.0249-25.2025.f1-4 Figure 1-4 **Comparison of Hand-Scoring and Automated EthoVision Tracking.** Results from hand-scoring and automated EthoVision tracking for one hour of time on nest (**A-C**) and maternal fragmentation (**D-F**). (**A**) Hand-scored values for dams’ time on nest (F(5,5) = 2.84, P = 0.84). (**B**) EthoVision-derived time on nest values for the same period (F(5,5) = 1.48, P = 0.57). (**C**) Correlation between hand-scored and EthoVision values for time on nest (CTL and LB combined; r = 0.83, R² = 0.69, P = 0.0008). (**D**) Hand-scored values for dams’ nest entries and exits (F(5,5) = 10.23, P = 0.06). (**E**) EthoVision fragmentation values for the same period (F(5,5) = 203.4, P = 0.027). (**F**) Correlation between hand-scored and EthoVision fragmentation values (CTL and LB combined; r = 0.37, R² = 0.14, P = 0.24). N = 6 litters per condition. Welch’s t-tests were used for panels A, B, D, and E, Pearson correlations for panels C and F. Download Figure 1-4, TIF file.

**Movie 1.** CTL cage postnatal day 3, dark phase. [View online]

**Movie 2.** LB cage postnatal day 3, dark phase. [View online]

**Movie 3.** LB cage postnatal day 3, dark phase, dam drugging a pup while nursing. [View online]

A 2 × 2 repeated-measures ANOVA (rmANOVA) found a significant interaction between pre- and postassignment conditions and rearing conditions for hourly distance traveled, attributed to significantly greater distance traveled by LB dams relative to CTL dams during the postassignment period (P2–P7; *p* < 0.0001), but not during the preassignment period (P0–P2, *p* = 0.14; Fig. 1*D*). Comparisons in distance traveled between pre- and postassignment found no significant changes in CTL dams (*p* = 0.39) as opposed to ∼50% increase during postassignment in LB dams (*p* < 0.0001; Fig. 1*D*). To further examine the effects of rearing on hourly distance traveled during the postassignment period, we conducted a three-way rmANOVA to characterize the effects of days and the dark/light phase as within-subjects variables and rearing as a between-subjects variable. This analysis revealed significant effects of day, rearing, the light–dark cycle, and a rearing by light–dark interaction (Fig. 1*E*). The effect of day was due to a rapid decline in distance traveled from P2 to P3, as dams adjusted to the bedding change. From P3–7, both CTL and LB dams’ locomotor activity exhibited a distinct diurnal rhythm, with higher activity during the dark phase (active phase for mice) than the light phase (Fig. 1*E*). This diurnal effect was greater for LB dams than CTL (Extended Data Fig. 1-1*A*).

A similar pattern was seen for maternal fragmentation, which is the frequency by which dams entered and exited the nest (Fig. 1*F*,*G*). Specifically, maternal fragmentation in CTL dams did not change after condition assignment (*p* = 0.49), whereas it was increased approximately threefold in LB dams (*p* < 0.0001; Fig. 1*F*). As with distance traveled, dams’ entrances/exits from the nest stabilized by P3 but was significantly higher in LB dams, especially during the dark phase (Fig. 1*G*, Extended Data Fig. 1-1*B*). LB, but not CTL, dams exhibited a diurnal rhythm in maternal fragmentation (light vs dark for CTL: *p* = 0.26, for LB: *p* < 0.0001; Extended Data Fig. 1-1*B*).

Next, we evaluated the effects of pre- versus postassignment and rearing on time in the nest. Both CTL and LB dams decreased the average time spent on the nest from pre- to postassignment; however, this reduction was greater for CTL than LB dams (CTL *p* < 0.0001, LB *p* = 0.0015), leading to LB dams spending more time than CTL on the nest from P2–P7 (CTL vs LB *p* = 0.004; Fig. 1*H*). Dams spent more time on the nest during the light (inactive) phase than during the dark (active) phase; as with movement and fragmentation, the difference between CTL and LB was greater during the dark phase (Extended Data Fig. 1-1*C*).

The effects of LB on dams’ movement and fragmentation were highly consistent across all three cohorts (Extended Data Fig. 1-2*A,B*), underscoring the reproducible nature of these measurements. In contrast, the effect of rearing on time in nest was more variable and was significant for Cohorts 1 and 2, but not in Cohort 3 (Extended Data Fig. 1-2*C*). Finally, comparable intragroup variances were found between CTL and LB dams for distance traveled (Extended Data Fig. 1-3*A*) and time in the nest (Extended Data Fig. 1-3*B*), but not for maternal fragmentation for which intersubject variability was significantly higher in LB dams (Extended Data Fig. 1-3*C*).

To assess the accuracy of automated tracking, a subset of 12 litters (*N* = 6 per rearing condition) were randomly selected for hand-scoring during 1 h of the dark phase, when low contrast is most likely to cause tracking errors. Across the 12 h assessed, EthoVision produced detection errors in two litters. In one CTL litter, EthoVision failed to track the dam when she was outside the nest for 430 s. Because this “missing” time was classified as nest time, total nest time was overestimated for this dam by 12%. Conversely, in one LB litter, EthoVision incorrectly tracked a location outside the nest for 768 s, resulting in a 21% underestimation of time on nest for that dam. Overall, hand-scored and automated time on nest values showed excellent agreement (*R*^2^ = 0.69, *p* = 0.0008; Extended Data Fig. 1-4*A–C*). However, this was not the case for the frequency of dam entrances and exits from the nest. Although the relative differences between CTL and LB values were consistent across both methods, hand-scored frequencies were lower than those generated by EthoVision, and the two measures were not significantly correlated (*R*^2^ = 0.17, *p* = 0.19; Extended Data Fig. 1-4*D–F*). This discrepancy was due to EthoVision detecting and scoring events in which the dam’s head or body crossed the nest boundary without fully exiting. As shown in Movie 5, these partial exits were primarily driven by stretch-attend postures, a defensive risk assessment behavior typically observed under uncertain conditions (Grewal et al., 1997; Holly et al., 2016). These “pseudo-exits” were not considered full sorties and were therefore excluded from hand scoring. Thus, EthoVision’s definition of fragmentation captures a broader set of aberrant maternal behaviors—including stretch-attend postures—in which the dam explores the surroundings without fully leaving the nest.

### Dam’s locomotor activity during the first week of life negatively correlated with pup bodyweight throughout development

A two-way ANOVA assessing the effects of rearing and sex on body weight at P8, P14, P18, P26, and P33 revealed significant and consistent lower body weight in LB pups compared with CTL from P8 to P33 (Fig. 2*A*), with similar outcomes obtained in Cohorts 2 and 3 (Extended Data Fig. 2-1). A transient, low-magnitude sex difference was noted at P14 but not at P18, with significant rearing × sex interactions detected at P26 and P33. These interactions were attributed to lower body weight in LB males, but not LB females, compared with their same-sex control groups (Fig. 2*A*). Mean pup body weight for each litter was inversely correlated to the dam’s average movement from P2–P7 (Fig. 2*B–F*), a finding that was consistent with previous research utilizing standardized home cage and aluminum flooring (Rice et al., 2008; Treccani et al., 2021). This inverse correlation was strongest at P8 (*R*^2^ = 0.76, *p* < 0.001) but persisted at P14 (*R*^2^ = 0.52, *p* = 0.019; Fig. 2*C*) and showed a trend at P18 (*R*^2^ = 0.27, *p* = 0.08; Fig. 2*D*). Due to the significant interaction between rearing and body weight at P26 and P33, separate analyses were conducted for males and females at these ages. While both male and female pups’ weights had significant negative correlation with the dams’ movement at P26 (Fig. 2*E*), at P33 this relationship was only significant in males (Fig. 2*F*). Although the dams’ movement and maternal fragmentation positively correlated (*R*^2^ = 0.36, *p* = 0.039, *N* = 12 litters), distance traveled provided a more reliable correlation with pup weight. These data reveal a strong negative and lasting relationship between the quality of maternal care during the first week of life, specifically distance traveled, and body weight that is more persistent in males.

**Figure 2.**
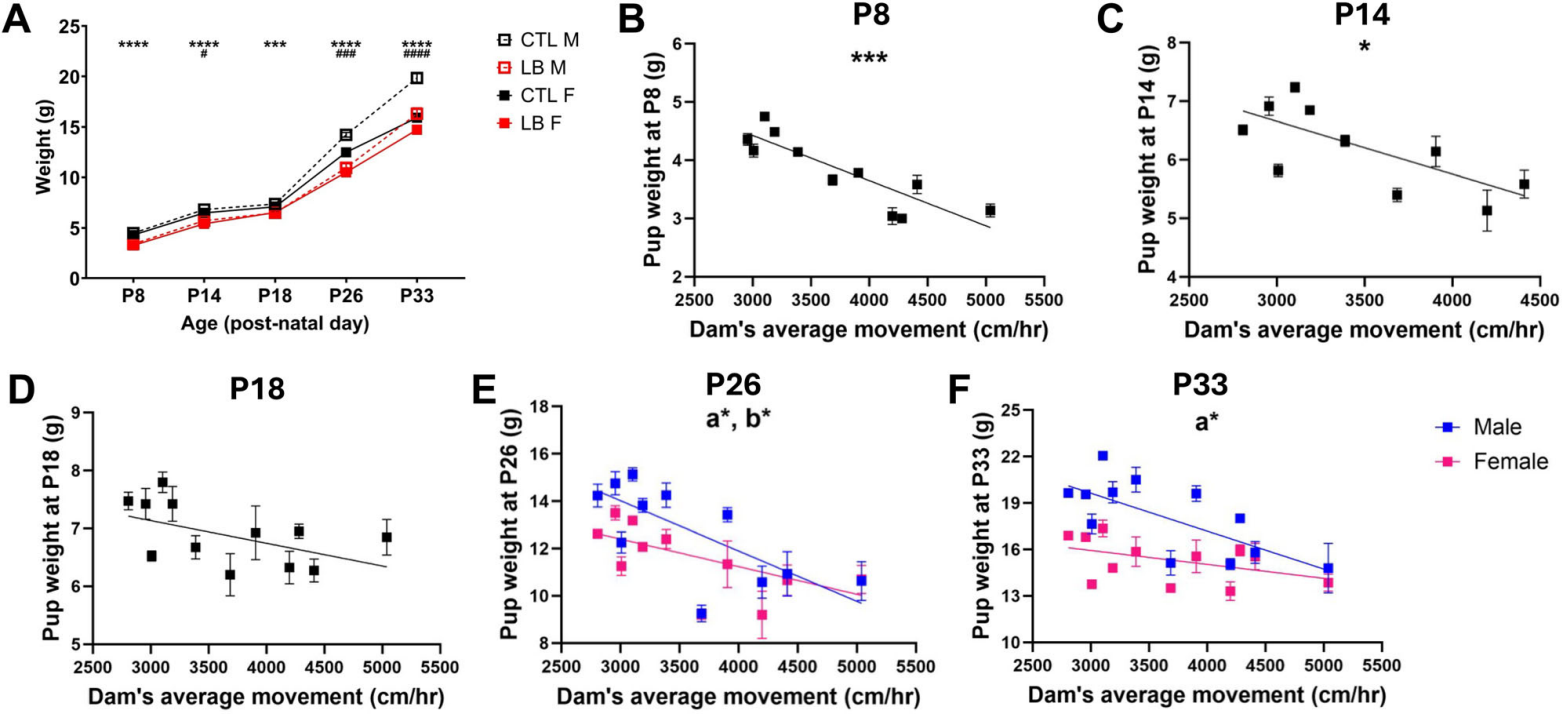
Dam’s locomotor activity during the first week negatively correlates with pup bodyweight throughout development. ***A***, Effects of rearing and sex on pup weights at P8, P14, P18, P26, and P33; * indicates effect of rearing, ^#^ indicates effect of sex. P8: rearing *F*_(1,72)_ = 153.4, *p* < 0.0001; sex *F*_(1,72)_ = 3.68, *p* = 0.059; interaction: NS. P14: rearing *F*_(1,66)_ = 53.01, *p* < 0.0001; sex *F*_(1,66)_ = 4.60, *p* = 0.036; interaction: NS. P18: rearing *F*_(1,44)_ = 14.75, *p* = 0.0004; sex: NS; interaction: NS. P26: rearing *F*_(1,74)_ = 75.98, *p* < 0.0001; sex *F*_(1,74)_ = 13.13, *p* = 0.0005; interaction *F*_(1,74)_ = 4.514, *p* = 0.037. P33: rearing *F*_(1,44)_ = 25.89, *p* < 0.0001; sex *F*_(1,44)_ = 35.16, *p* < 0.0001; interaction *F*_(1,44)_ = 6.42, *p* = 0.015. Correlation between pup body weight at P8 and dams’ average movement P2–7 at different ages. ***B***, P8: *R*^2^ = 0.76, *p* = 0.0005. ***C***, P14: *R*^2^ = 0.52, *p* = 0.019. ***D***, P18: *R*^2^ = 0.27, *p* = 0.08. ***E***, P26: males: *R*^2^ = 0.49, *p* = 0.016, females: *R*^2^ = 0.42, *p* = 0.030. ***F***, P33: males: *R*^2^ = 0.50, *p* = 0.01, females: *R*^2^ = 0.20, *p* = 0.14. Two-way ANOVA for each time points for ***A***, with *N* = 4–6 litters, and *N* = 9–23 pups per rearing condition per time point. Pearson’s correlation for ***B–F*** for CTL (black) and LB (red) litters. Male and female pups were analyzed separately in ***E***, ***F*** (male: open symbols, dotted line, labeled lower case “a”; female: closed symbols, solid line, labeled lower case “b”). NS, nonsignificant, *p* > 0.05. See Extended Data Figure 2-1 for more details.

10.1523/ENEURO.0249-25.2025.f2-1 Figure 2-1 **Pup Weights at P7 and P13.** Pup weights from cohorts 2 **(A)** and 3 **(B)**. **(A)** LB pups weigh significantly less than CTL pups at P7 without an effect of sex. N = 24-40 per group, from N = 9 litters per rearing condition. Rearing: F(1, 124) = 74.84, P < 0.0001; Sex: F(1, 124) = 1.227, P = 0.27; Interaction: F(1, 124) = 0.0046, P = 0.95. **(B)** LB pups weigh significantly less than CTL pups at P13 without an effect of sex. N = 13-20 per group, from N = 6 CTL and 5 LB litters. Rearing: F(1, 64) = 101.9, P < 0.0001; Sex: F(1, 64) = 0.79, P = 0.38; Interaction: F(1, 64) = 0.017, P = 0.90. Analysis by 2-way ANOVA. Download Figure 2-1, TIF file.

### LB increased corticosterone levels in P7 pups

Previous work in mice has found a negative correlation between maternal fragmentation and corticosterone levels in P9 pups (Rice et al., 2008; Treccani et al., 2021) and similar findings in rats causally linked elevated corticosterone during the first week in life with abnormal attachment-like behavior in LB pups (Raineki et al., 2019; Opendak et al., 2020). Therefore, in a separate cohort of litters, we tested the dam and pups' baseline blood corticosterone levels at P7 (Fig. 3*A*; for maternal behavior in this cohort, see Extended Data Fig. 1-2). Corticosterone levels in LB pups were more variable and significantly higher in LB than CTL pups, with no differences between males and females (Fig. 3*B*). Dams' corticosterone levels were comparable between CTL and LB conditions (Fig. 3*C*) with no significant correlation between corticosterone levels in the dam and her pups (CTL: *r* = 0.48, *p* = 0.34; LB: *r* = 0.62, *p* = 0.19; *N* = 6 dams per rearing condition, and 3–4 pups per litter). In agreement with previous reports (Rice et al., 2008; Treccani et al., 2021), we found a significant correlation between maternal fragmentation and corticosterone levels in P7 pups (Fig. 3*D*).

**Figure 3.**
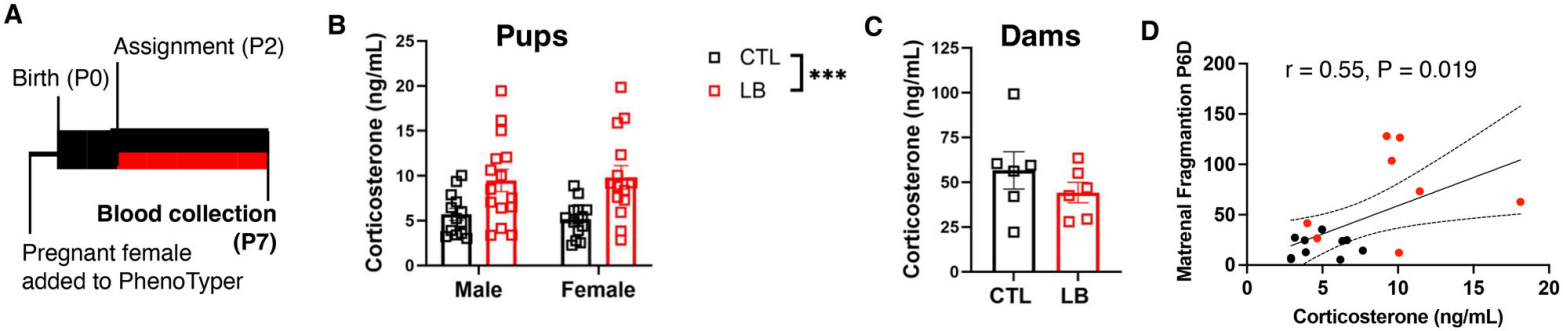
LB increases corticosterone levels in P7 pups. ***A***, Experimental timeline. Boxes indicate recording periods, and lines indicate nonrecording periods. Black denotes CTL conditions, and red denotes LB conditions. ***B***, LB pups have higher, but more variable, corticosterone levels than CTL pups at P7; *N* = 13–15 pups per sex per rearing condition from a total of 9 litters per condition. Two-way ANOVA, rearing: *F*_(1,52)_ = 17.61, *p* = 0.0001; sex: NS; interaction: NS. ***C***, No difference between dams' corticosterone levels at P7. Unpaired *t* test, *t*_(10)_ = 1.044, *p* = 0.32; *N* = 6 dams per condition. ***D***, Maternal fragmentation during the dark phase of P6 (prior to corticosterone assessment) correlated with the average corticosterone levels per liter obtained at P7, *R*^2^ = 0.30, *p* = 0.019, *N* = 2–4 pups per litter with nine litters per condition. NS, nonsignificant, *p* > 0.05.

### LB pups had reduced vocalizations at P8 but equivalent maternal buffering

A critical aspect of maternal attachment is the ability of the dam to reduce pups' distress, a phenomenon known as “maternal buffering” (Bowlby, 1978; Kikusui et al., 2006; Gunnar et al., 2015). Here we assessed the effects of sex and rearing on maternal buffering in P8 pups (Fig. 4*A*; see also Extended Data Fig. 1-2 for maternal behavior of Cohort 1) by comparing the number of ultrasonic vocalizations (USVs) emitted by pups in response to a brief 5 min separation from the dam followed by a reunion with an anesthetized “aunt” (Fig. 4*B*). We used anesthetized unrelated adult female instead of the dam in order to avoid exposing pups to anesthetic in the breast milk and because preliminary work from our lab and other groups has shown that at this age pups respond similarly to their dam and an unfamiliar aunt as long as they are fed the same diet (Opendak et al., 2020). A three-way rmANOVA assessing the effects of test phase (i.e., isolation vs with aunt), rearing, and sex demonstrated significant main effects of phase and rearing as well as significant interaction between phase and rearing and phase and sex (Fig. 4*C*). Post hoc analysis during the isolation phase revealed significant effect of rearing due to reduced vocalization in LB compared with CTL, with no significant effect of sex or interaction. No significant effects of rearing, sex, or interaction between sex and rearing were found for the with aunt phase (Fig. 4*C*). Body weight did not correlate with frequency of USVs during isolation (CTL: *R*^2^ = 0.0053, *p* = 0.67, LB: *R*^2^ = 0.017, *p* = 0.43; Extended Data Fig. 4-1*A*), indicating that the reduced USVs observed in LB pups were not mediated by prematurity or low body weight. Furthermore, there was no significant correlation between maternal care and USVs during isolation in LB pups (distance traveled: *r* = −0.067, *p* = 0.89, fragmentation: *r* = −0.15, *p* = 0.78; Extended Data Fig. 4-1*B*). Reunion with the aunt led to a robust buffering effect in all groups, with no significant effects of rearing, sex, or interaction (Fig. 4*D*). Together, these data indicate that LB reduces distress vocalizations in response to maternal separation but does not disrupt maternal buffering.

**Figure 4.**
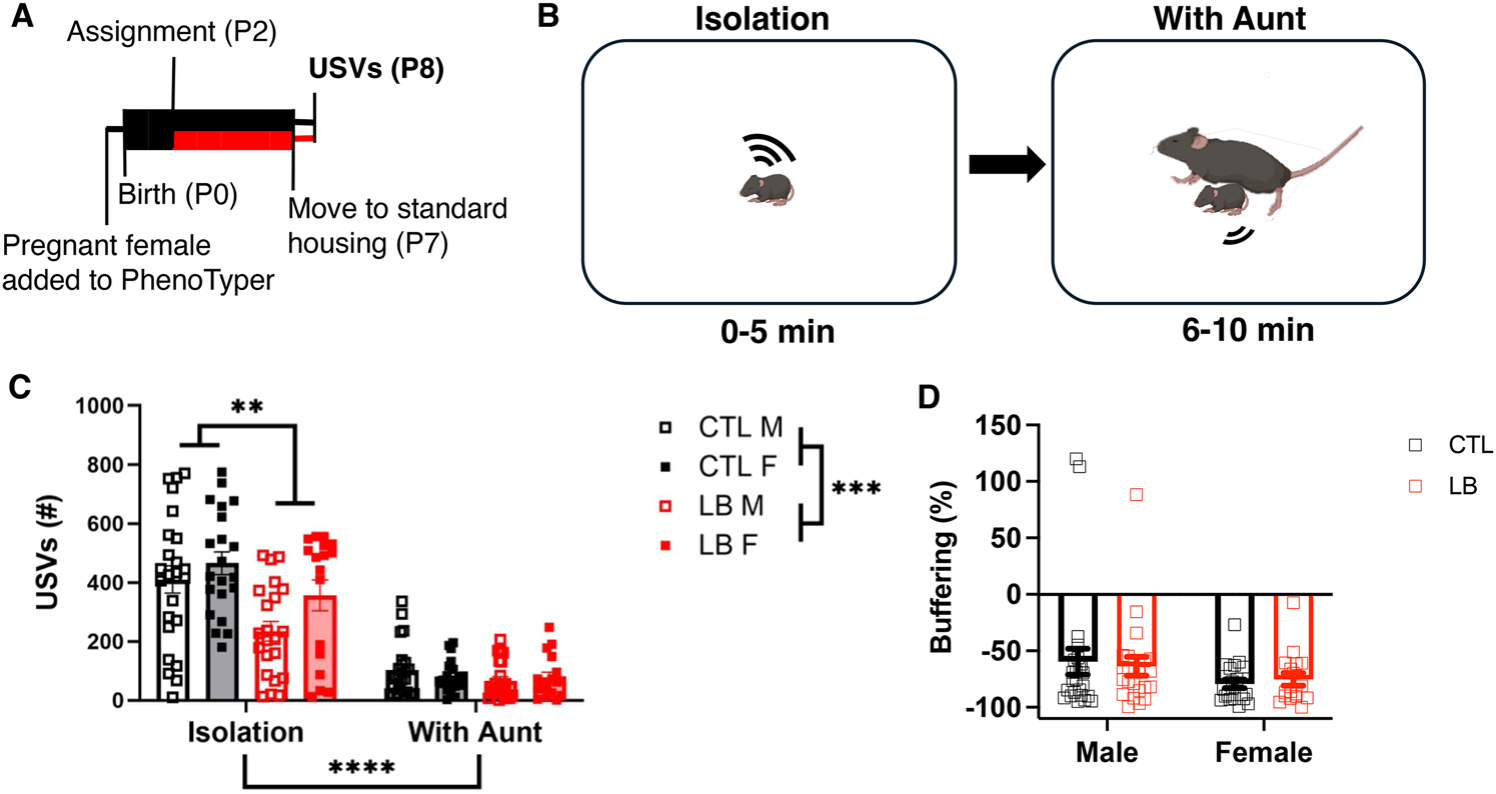
LB pups have reduced vocalization during isolation but do not differ in buffering at P8. ***A***, Timeline. Boxes indicate recording periods, and lines indicate nonrecording periods. Black denotes CTL conditions, and red denotes LB conditions. ***B***, Schematics of the test. ***C***, Total number of USVs during the two test phases. rmANOVA of isolation phase only, rearing: *F*_(1,72)_ = 8.276, *p* = 0.0053; sex: *F*_(1,72)_ = 3.68, *p* = 0.059; interaction: NS. rmANOVA of reunion phase only: rearing, sex and interaction are all NS. ***D***, Percent change in vocalizations between phases (buffering). 2 × 2 ANOVA, rearing: NS; sex: *F*_(1,80)_ = 3.34, *p* = 0.071; interaction: NS. *N* = 5–6 litters and *N* = 17–24 pups per group. NS, nonsignificant, *p* > 0.05. See Extended Data Figure 4-1 for more details.

10.1523/ENEURO.0249-25.2025.f4-1 Figure 4-1 **P8 Isolation USVs Do Not Correlate to Pup Weight or Dam Behavior. (A)** Correlation between P8 CTL and LB pup weights and number of USVs during the isolation phase of the maternal buffering test. N = 37-39 per condition, sexes are combined for analysis since neither behavior nor weight varied by sex. CTL: R^2^ = 0.0053, P = 0.67; LB: R^2^ = 0.017, P = 0.43. **(B)** Dams’ average movement P2-7 does not correlate with pups’ USVs during isolation at P8. CTL: R^2^ = 0.36, P = 0.29; LB: R^2^ = 0.03, P = 0.74. Download Figure 4-1, TIF file.

### LB impaired proximity-seeking behavior in P13 pups

Using a proximity-seeking approach toward an anesthetized dam as a measure of attachment-like behavior, Raineki et al. (2019) demonstrated that LB impairs approach behavior in P13 rat pups. These deficits were directly linked to elevated corticosterone levels in LB pups (Raineki et al., 2019). Given that LB increased corticosterone levels in P7 pups (Fig. 3*B*), we tested whether similar deficits are observed in a separate cohort of P13 LB mice pups and whether they correlated with abnormal maternal care (Fig. 5*A*,*B*; Extended Data Fig. 1-2 for maternal behavior in Cohort 3). Ninety percent of all CTL pups (35/39) approached and maintained close proximity to the dam. In contrast, only 48% of LB pups (14/29) approached the dam (*χ*^2^_(1,14.2)_ = 3.77, *p* = 0.0002; Fig. 5*C*), with no significant correlation between the proportion of pups that approached the dam within each litter and the dam’s average movement (CTL: *r* = −0.69, *p* = 0.13; LB: *r* = 0.54, *p* = 0.35). Similar results were obtained using the average distance maintained between the pup and the dam (*p* = 0.0001; Fig. 5*D*). Within each LB litter, approximately half of the pups approached the dam while the remaining pups stayed in place or moved away from the dam (Movie 4), with no differences in weight, sex, or order of testing between LB pups that approached versus those that did not approach the dam (Extended Data Fig. 5-1). For the LB pups that did approach the dam, there was a trend toward longer latency to approach (rearing: *F*_(1,45)_ = 3.91, *p* = 0.054; Fig. 5*E*), but no difference in total interaction time (Fig. 5*F*). In contrast to findings by Raineki et al., CTL and LB pups did not differ in time spent on the dam’s dorsal or ventral side (*F*_(1,45)_ = 0.12, *p* = 0.73). We replicated these results in an independent cohort raised in standard cages (Extended Data Fig. 5-2), demonstrating that the findings are robust and not dependent on raising pups in the PhenoTyper. In summary, LB reduces the likelihood of proximity-seeking behavior toward an anesthetized dam in P13 mice and rats. However, unlike findings in rats, LB did not alter the nature of the interaction in mice pups that engage with the dam, and the reduction in approach behavior was driven by notable individual differences within a litter.

**Figure 5.**
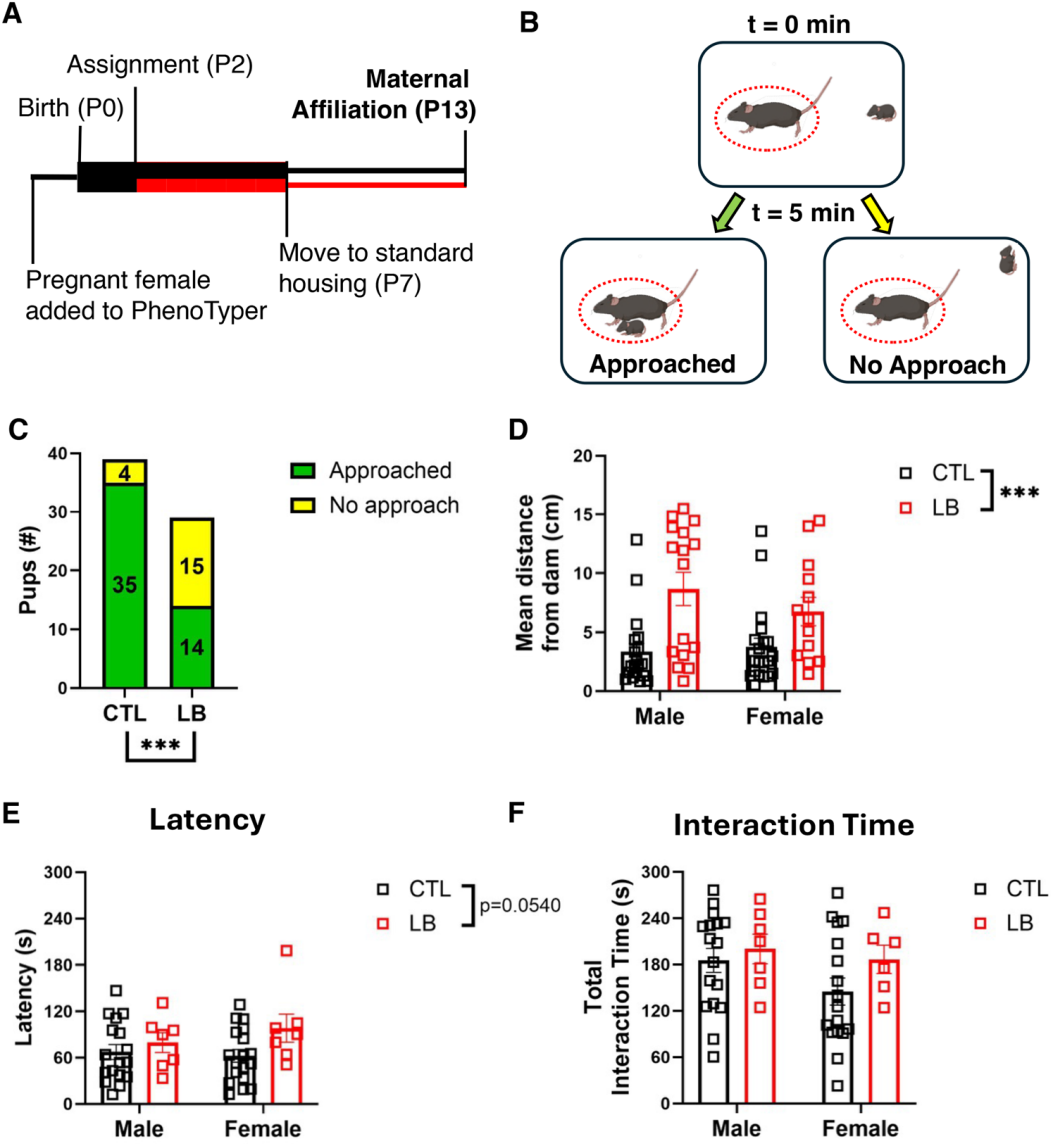
LB impairs approach behavior in P13 pups. ***A***, Timeline. Boxes indicate recording periods, and lines indicate nonrecording periods. Black denotes CTL conditions, and red denotes LB conditions. ***B***, Experimental setup. Dotted red line indicates interaction zone. ***C***, 35 out of 39 (90%) of CTL pups approached the dam versus 14 out of 29 (48%) of LB pups: *χ*^2^_(1,14.2)_ = 3.77, *p* = 0.0002. ***D***, Average distance from the dam. Rearing: *F*_(1,64)_ = 17.11, *p* = 0.001; sex: NS; interaction: NS. ***E***, Latency to approach the dam among pups that did so. Rearing: *F*_(1,45)_ = 3.92, *p* = 0.054; sex: NS; Interaction: NS. ***F***, Total interaction time among pups that approached the dam. Rearing, sex, and interaction were all NS. Two-way ANOVA in ***D***, ***E***, and ***F***. *N* = 13–20 per sex and rearing condition (total of 39 CTL and 29 LB pups), from *N* = 6 CTL and 5 LB litters. NS, nonsignificant, *p* > 0.05. See Extended Data Figures 5-1 and 5-2 for more details.

10.1523/ENEURO.0249-25.2025.f5-1 Figure 5-1 **Effects of Body Weight, Sex, and Order of Testing on Approach behavior in P13 LB pups**. **(A)** Correlation between pup body weight and latency to reach the dam for CTL and LB pups that approached. CTL: R^2^ = 0.075, P = 0.11; LB: R^2^ = 0.037, P = 0.51. **(B)** Body weight, t(27) = 0.98, P = 0.34. **(C)** Sex, Fisher’s exact test, P = 0.72. **(D)** Testing order. Fisher’s exact test, P = 0.83. N = 29 pups from N = 5 litters, sexes combined for A & C. Download Figure 5-1, TIF file.

10.1523/ENEURO.0249-25.2025.f5-2 Figure 5-2 **Similar P13 Results in a Cohort of Standard-housed Pups. (A)** 19 out of 21 (90%) of CTL pups approached the dam vs. 7 out of 29 (24%) of LB pups: Fisher’s exact test P < 0.0001. **(B**) Average distance from the dam. Rearing: F (1, 46) = 32.42, P < 0.0001; Sex: NS; Interaction: NS. **(C)** Total interaction time among pups that approached the dam. Rearing, sex and interaction were all NS. **(D)** Latency to approach the dam among pups that did so. Rearing, sex and interaction were all NS. 2-way ANOVA in B, C, and D. N = 10-19 per sex and rearing condition (total of 21 CTL and 29 LB pups), from N = 3 CTL and 4 LB litters. NS-non-significant, P > 0.05. Download Figure 5-2, TIF file.

**Movie 4.** Maternal affiliation test of postnatal 13 CTL (right) and LB (left) pups. Approximately half of all LB pups did not approach or sought proximity with their anesthetized dam versus 10% of CTL pups that displayed this avoidant behavior. [View online]

### LB pups had higher anxiety-like behavior but spent equivalent time with the dam at P18

Attachment to a caregiver is thought to reduce anxiety and provide a secure base for exploring the environment. We therefore examined the effects of rearing and sex on exploratory behavior in P18 pups (Fig. 6*A*,*B*). CTL and LB pups were equally active exploring the empty arena (Fig. 6*C*), indicating the same degree of mobility despite LB pups’ lower body weight (Fig. 2*A*). However, LB pups spent approximately half the amount of time in the center arena as their CTL counterparts (Fig. 6*D*), indicating a higher level of anxiety-like behavior. Time in center did not correlate to the dams' movement P2–7 (CTL: *R*^2^ = 0.05, *p* = 0.71; LB: *R*^2^ = 0.59, *p* = 0.13) and a similar difference in anxiety-like behavior was observed in CTL and LB mice raised in standardized cages instead of the PhenoTyper (Extended Data Fig. 6-1*A,B*). Immediately following the open field test, maternal preference was tested by quantifying the time spent in the vicinity of the dam or an inanimate object. Because the dam was not anesthetized for this test, dam movement and direct contact were restricted by placing her and the object under inverted wire cups in the open field arena. All groups spent more time in the vicinity of the dam compared with the novel object, with no significant effects of rearing, sex, or interaction (Fig. 6*E*). Maternal preference, calculated as the percent time spent in vicinity of dam over total time spent with either dam or the inanimate object, was significantly greater than 50% in all groups (*p* < 0.0001) indicating robust preference toward the dam with no significant effects of rearing, sex, or interaction (Fig. 6*F*). Similar findings were observed in an independent cohort raised in standard cages (Extended Data Fig. 6-1*C,D*). Thus, although LB pups have increased anxiety-like behavior at P18, they have normal maternal preference.

**Figure 6.**
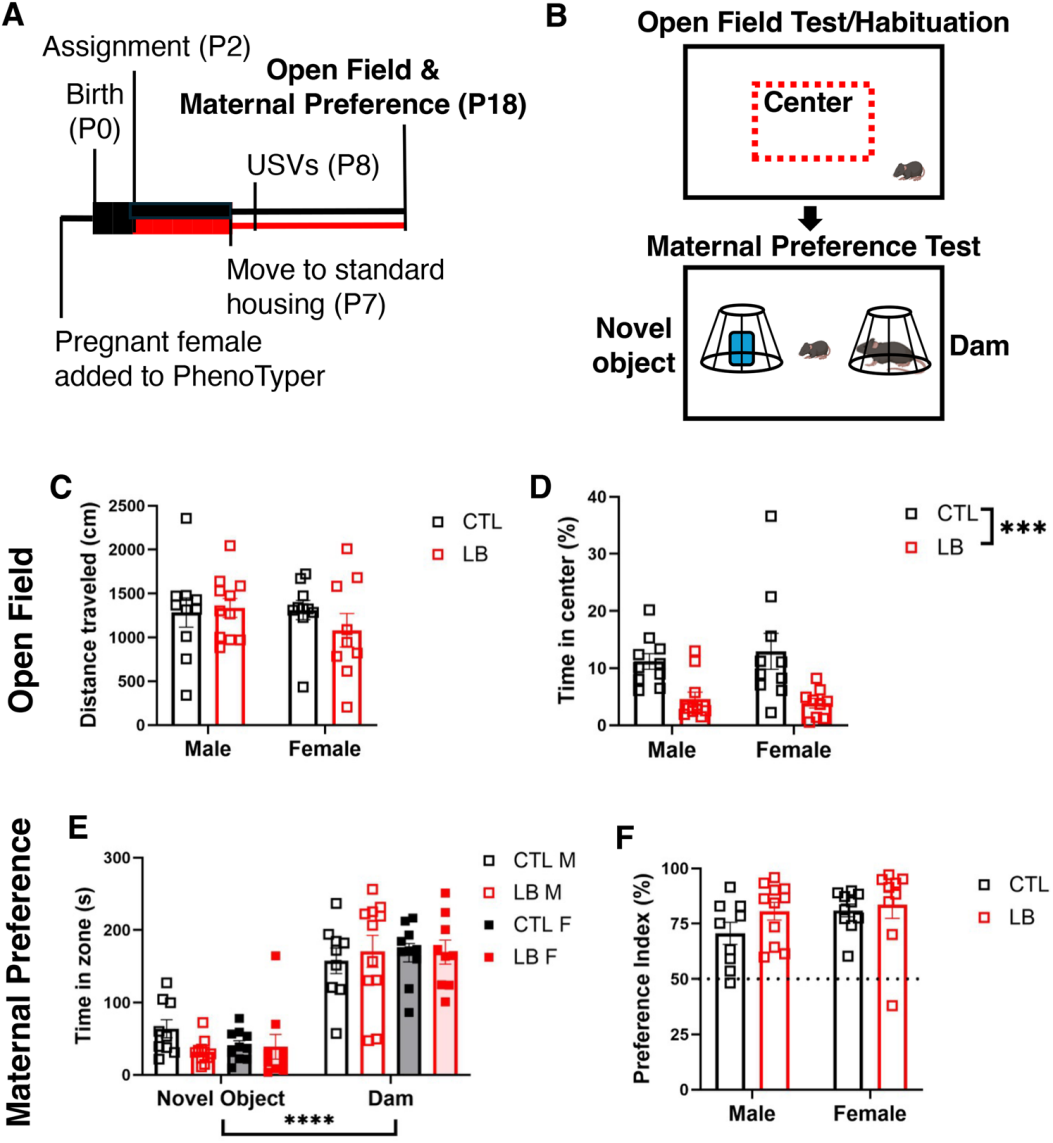
LB pups show more anxiety-like behavior but equivalent maternal preference at P18. ***A***, Experimental timeline. Boxes indicate recording periods, and lines indicate nonrecording periods. Black denotes CTL conditions, and red denotes LB conditions. ***B***, Schematic of open field and maternal preference tests. ***C***, Distance moved during the open field test. Rearing, sex, and interaction were all NS. ***D***, Percent time spent in the center of the arena. Rearing: *F*_(1,36)_ = 17.29, *p* = 0.0002; sex: NS; interaction: NS. ***E***, Time spent near the novel object and the dam. Zone: *F*_(1,70)_ = 137.4, *p* < 0.0001; rearing, sex, and all interactions were NS. ***F***, Preference index. Rearing, sex, and interactions were all NS. *N* = 5–6 litters and *N* = 9–11 pups per group. 2 × 2 ANOVA in ***C***, ***D***, and ***F***. Three-way rmANOVA in ***E***. NS, nonsignificant, *p* > 0.05. See Extended Data Figure 6-1 for more details.

10.1523/ENEURO.0249-25.2025.f6-1 Figure 6-1 **Similar P18 Results in a Cohort of Standard-housed Pups. (A)** Distance moved during the open field test. Rearing: F (1, 50) = 2.39,P = 0.13; Sex: F (1, 50) = 0.37, P = 0.55; Interaction: F (1, 50) = 2.56, P = 0.12. **(B)** Time spent in the center of the arena. Rearing: F (1, 50) = 11.10, P = 0.0016; Sex: F (1, 50) = 0.0076, P = 0.93; Interaction: F (1, 50) = 1.25, P = 0.27. **(C)** Time spent near the novel object and the dam. Zone: F (1, 37) = 116.5, P < 0.0001; Rearing: F (1, 37) = 2.19, P = 0.15; Sex: F(1, 37) = 0.65, P = 0.42; Zone x Sex: F(1, 37) = 0.1, P = 0. 32; Zone x Rearing: F (1, 37) = 2.81, P = 0.10; Sex x Rearing: F (1, 37) = 0.41, P = 0.52; Zone x Sex x Rearing: F (1, 37) = 0.033, P = 0.86. **(D)** Preference index. Rearing: F (1, 37) = 0.057, P = 0.81; Sex: F (1, 37) = 0.26, P = 0.62; Interaction: F (1, 37) = 0.26, P = 0.61. N = 9-12 pups per group from N = 4-5 litters. 2 × 2 ANOVA in A, B, & D. 3-way rmANOVA in C. Download Figure 6-1, TIF file.

### No effect of LB on juvenile social interaction

According to attachment theory, deficits in attachment to a care giver also impact social affiliation later in life. Moreover, studies in both rats and mice have reported abnormal social interaction and play behavior in rodents raised under LB conditions that appear to be more prominent in males (Molet et al., 2016; Bolton et al., 2018; Birnie et al., 2023). Therefore, we tested social interaction at P33–34 with an age- and sex-matched CTL-raised mouse and analyzed interactions initiated by the experimental mouse (Fig. 7*A*,*B*). There were no differences between CTL and LB in latency to first interaction (Fig. 7*C*) or cumulative time spent interacting (Fig. 7*D*), nor did these metrics correlate to the dams' movement from P2 to P7 (Latency: CTL: *R*^2^ = 0.0088, *p* = 0.86. LB: *R*^2^ = 0.13, *p* = 0.48. Total interaction time: CTL: *R*^2^ = 0.039, *p* = 0.71, LB: *p* = 0.31, *R*^2^ = 0.25).

**Figure 7.**
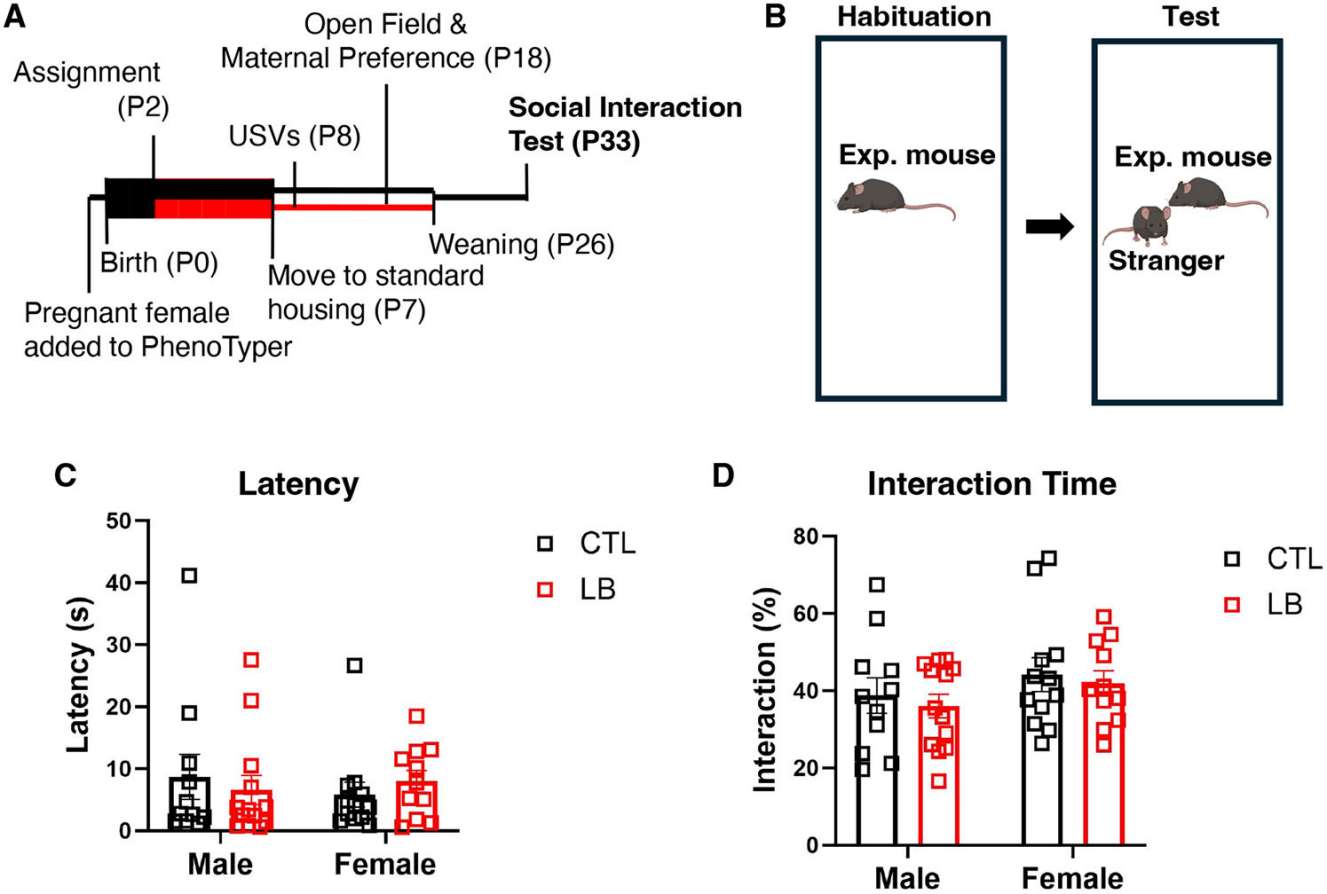
CTL and LB pups have equivalent social interaction. ***A***, Timeline. Boxes indicate recording periods, and lines indicate nonrecording periods. Black denotes CTL conditions, and red denotes LB conditions. ***B***, Test schematic. ***C***, Latency to first social contact initiated by the experimental mouse. Rearing, sex, and interaction were all NS. ***D***, Cumulative time that the experimental mouse interacted with the stranger, expressed as a percent of test time. Rearing, sex, and interaction were all NS. *N* = 6 litters and *N* = 11–13 pups per group. 2 × 2 ANOVA. NS, nonsignificant, *p* > 0.05.

## Discussion

Despite its conceptual and clinical promise, little is currently known about the underlying biology of attachment formation in humans, whether under normal or adverse conditions. However, the conserved nature of attachment behavior across diverse species, including rodents, suggests that rodent models may offer valuable insights (Sullivan and Opendak, 2021). Elegant work in rats has demonstrated that dams in poverty-like conditions, characterized by limited bedding and nesting (LB), exhibit erratic and abusive-like behaviors during the postnatal period (Walker et al., 2017). This abnormal form of maternal care is associated with elevated corticosterone levels in the pups (Rice et al., 2008; Raineki et al., 2019; Treccani et al., 2021), and the repeated association between maternal cues and corticosterone elevation leads to reduced cortical responsiveness to nurturing maternal care (Opendak et al., 2020), as well as abnormal amygdala development (Raineki et al., 2019). Furthermore, activation of the amygdala in response to maternal cues is directly responsible for deficits in attachment-like behavior observed in LB rat pups (Raineki et al., 2019).

To the best of our knowledge, the effects of LB on maternal attachment behavior and the associated neurological correlates have not yet been investigated in mice, where additional genetic tools can be leveraged to clarify the underlying biology. Moreover, although several studies have used continuous monitoring to characterize the impact of LB on maternal behavior in mice (Gallo et al., 2019; Demaestri et al., 2022), none have attempted to directly link these maternal behavioral abnormalities with deficits in attachment-like behaviors. The primary objective of this study was to determine whether LB induces attachment-like deficits in mice by employing multiple behavioral tests (e.g., maternal buffering at P8, maternal affiliation at P13, and maternal preference at P18). In addition, we used a 24/7 automated home-cage monitoring system to more precisely characterize maternal care and to examine how these alterations relate to attachment-like deficits. To achieve this, we utilized the PhenoTyper system, which enables unbiased and automated quantification of maternal behavior throughout the light/dark cycle without investigator interference (Mingrone et al., 2020). Using three independent cohorts composed of a total of 21 CTL and 20 LB litters, we confirmed that LB conditions consistently increased the total movement of dams by ∼50% and fragmentation of care by 300% compared with dams in CTL conditions. These results confirm a substantial body of previous research indicating that LB increases maternal fragmentation (Walker et al., 2017; Gallo et al., 2019; Demaestri et al., 2020; Demaestri et al., 2022) and strengthens these conclusions by utilizing substantially longer analysis periods.

Average time spent on the nest decreased from pre- to postassignment for both groups across all cohorts (Fig. 1*H*), suggesting that as pups mature, they require less maternal care. Overall, LB dams spent significantly more time on the nest, particularly during the dark phase (Fig. 1*H*,*I*). However, the relationship between rearing condition and time spent on the nest varied among the different cohorts. Specifically, LB dams spent more time on the nest in Cohorts 1 and 2, but there was no significant difference between the groups in Cohort 3 (Extended Data Fig. 1-2). This variability may account for the conflicting outcomes reported in the literature, with some studies indicating increased time spent in the nest by LB dams (Gallo et al., 2019; Granata et al., 2021; Treccani et al., 2021), while others found no differences between the groups (Rice et al., 2008; Molet et al., 2016). LB dams may spend more time on the nest because their pups are smaller and/or as a compensatory mechanism for their increased erratic and fragmented care. Excessive time spent on the nest may also elevate the rate of rough handling and stepping on pups, as documented in previous studies (Gallo et al., 2019; Raineki et al., 2019). In the present study, we found that LB elevated the frequency of stretch-attend postures (Movie 5, Extended Data Fig. 1-4), suggesting an increase in defensive-like behaviors while dams cared for pups in the nest (Grewal et al., 1997; Holly et al., 2016). Collectively, time spent in the nest is a more variable aspect of the LB paradigm that requires further characterization and assessment of its impact on attachment-like behaviors.

**Movie 5.** Typical stretch-attend posture in LB cage, postnatal day 3, dark phase. [View online]

The effects of LB on pup weight and development depend on the timing, duration, and severity of the manipulation (Rice et al., 2008; White and Kaffman, 2019; Demaestri et al., 2020). In this study, pup weight at P8 was strongly and inversely correlated with the dams’ movement from P2 to P7. This finding is consistent with previous work (Rice et al., 2008), but the magnitude of the correlation was higher when using continuous monitoring (*R*^2^ = −0.76, Fig. 2*B* vs *R*^2^ = −0.26 in Rice et al., 2008). Furthermore, the impact of the dam’s movement from P2 to P7 on body weight remained significant at P14, P18, and P26 for both sexes. This relationship continued to be significant for males but not for females at P33. These findings reveal a novel relationship between erratic maternal care early in life and long-term growth, which appears to be more persistent in male offspring. The mechanisms responsible for the reduced body weight observed in LB mice have yet to be clarified; however, Noviana et al. (2023) found that interventions aimed at increasing secure attachment were effective in reducing stunting in high-risk populations, demonstrating a potentially significant link between abnormal attachment and deficits in normal growth. We also replicated previous work showing that LB increased corticosterone levels in pups (Molet et al., 2014; Bath et al., 2016) and that this occurred in a manner which correlated with maternal fragmentation (Rice et al., 2008; Raineki et al., 2019; Treccani et al., 2021). No differences in corticosterone levels were found between CTL and LB dams suggesting that the elevated corticosterone levels in pups were not mediated through maternal transfer during suckling.

USVs at P8 are the murine equivalent of a baby’s cry; they serve as distress calls designed to elicit caregiving (Zimmer et al., 2019). Our findings indicate that P8 LB pups produced fewer USVs during isolation compared with CTL pups. This reduction was not correlated with decreased body weight or maternal fragmentation. A similar decrease in USVs was reported previously but only in female LB pups (Granata et al., 2021). Given that Agouti-related peptide (AgRP)-expressing neurons in the arcuate nucleus have been found to promote isolation-related USVs in mouse pups (Zimmer et al., 2019), it would be interesting to know whether LB inhibits AgRP activation in response to isolation. Since LB pups are accustomed to a poor nest environment and lower-quality maternal care, they may experience less distress during isolation than CTL pups. Alternatively, they may be less motivated to elicit retrieval by the dam due to her frequent departures from the nest and/or rough handling within the nest. Nevertheless, reunion with an aunt reduced USVs by ∼70% across all groups. Note that because buffering is reported as a percent change in USVs, normalized to the number of USVs during isolation for each pup, there is a floor effect by which reduction below 100% is not possible. Zimmer et al. (2019) found that warmth reduced AgRP-neuron activation during isolation in P10 mouse pups; thus, the body heat provided by contact with the aunt likely contributed to the buffering effect. Further experiments are needed to determine what sensory cues drive the buffering of USVs at P8. Regardless of which cues are used, the reduced USVs observed in response to separation in 8-d-old LB pups suggest an avoidant-like attachment style that is also highly responsive to a maternal presence.

Reduced rates of maternal approach observed at P13 among LB pups are consistent with findings in rats (Raineki et al., 2019). However, there are notable differences between our results and those reported in rats. For instance, Raineki and colleagues identified deficits in nipple attachment and relocation to the back of the dam in LB pups, whereas we primarily observed deficits in approach behavior. Furthermore, we observed substantial individual variability in approach behavior within LB litters, a finding that was replicated in an independent cohort of mice raised in standard cages. Although LB has been found to delay developmental parameters such as eye opening (Demaestri et al., 2020), there was no correlation between pup weight and latency to approach and no difference in weight between LB pups that did or did not approach. Nevertheless, we cannot rule out the possibility that LB delays some developmental process that guides this behavior. Aside from the use of mice instead of rats, there are three primary differences in experimental design that may explain the differences in our finding from Raineki et al. First, Raineki et al. only exposed litters to LB from P8 to P13, whereas we started LB at P2. Therefore, the pups in this study were subject to adverse conditions during more of their development (also resulting in lower body weight, which was not seen by Raineki et al.). Second, the maternal affiliation test by Raineki et al. did not use dirty bedding in the testing arena. However, we found that a small amount of dirty bedding was necessary to prevent the majority of pups from freezing. Finally, Raineki et al. tested pups for 30 min, whereas our test lasted for only 5 min. This shortened time was chosen to allow more pups to be tested per litter without extensive separation from the dam. However, it is possible that more CTL pups would have moved to the nipple and/or a greater proportion of LB pups would have approached the dam given more time. Additional studies are necessary to elucidate the underlying biology that drives these individual differences and to determine whether they represent stable long-term changes in other behavioral tests. The reduced rate of proximity-seeking in 13-d-old pups further supports the notion that LB causes avoidant-like attachment deficits.

In the open field test, 18-d-old LB pups spent less time exploring the center. Total distance traveled was unaffected, indicating that reduced center exploration was not due to changes in locomotor activity. Similar outcomes were observed in LB pups reared in the PhenoTyper (Fig. 6*C*,*D*) and in a separate cohort of mice raised in standard cages (Extended Data Fig. 6-1*A,B*). These results are consistent with our previous findings in juvenile mice (Johnson et al., 2018) and with reports of higher anxiety levels in children with insecure attachment (Feeney, 2000; Hornor, 2019; Gregory et al., 2020). Nevertheless, additional anxiety-like tests are required to determine whether this phenotype reflects primarily thigmotaxis in the open field or a broader increase in defensive behavior. For the effects of LB on anxiety-like behavior in nonattachment contexts, see Loi et al. (2017), Walker et al. (2017), and White and Kaffman (2019).

Both CTL and LB pups showed a strong preference for the dam over an inanimate object in the maternal preference test, indicating normal attachment-like behavior in this test. Similar results were obtained in CTL and LB litters raised under standard conditions (i.e., not in the PhenoTyper), indicating that these outcomes are robust and do not require specialized housing conditions. This test is unable to distinguish between the pup’s drive to interact with the dam specifically or a broader preference for social interaction. However, social drive is linked to maternal attachment (Bowlby, 1978; Izaki et al., 2024). Further studies will be needed to distinguish between social drive and maternal preference per se at this age. In P33–P34 juveniles, social exploration was comparable in CTL and LB mice during free interactions with a CTL-reared age- and sex-matched stranger, indicating that some attachment-like behaviors are resilient even in mice exposed to high levels of maternal fragmentation.

In conclusion, automated continuous home-cage monitoring provides a powerful method for quantifying maternal care in mice raised under CTL and LB conditions. Using this approach, we extended previous observations by demonstrating that differences in maternal behavior are more pronounced during the dark phase and that time spent on the nest is a more variable outcome. Maternal fragmentation, which included high levels of stretch-attend defensive postures, exhibited a strong negative correlation with body weight and resulted in stunted growth that was more persistent in males. LB pups displayed avoidant-like attachment deficits, as evidenced by reduced USVs in response to maternal separation at P8, deficits in approaching an anesthetized dam at P13, and decreased exploration of the center in the open field test at P18. This finding is consistent with clinical work linking childhood adversity, particularly neglect, with avoidant attachment (Mikulincer, 2016; Widom et al., 2018; Midolo et al., 2020; Tian et al., 2025) However, none of the observed deficits correlated with maternal behavior, suggesting a possible threshold effect rather than a graded effect. This hypothesis is keeping with the notion of the “good enough” parent, proposed by Donald Winnicott in the 1950s (Taylor et al., 2009). Intriguing within-litter individual differences in approach behavior were observed in both CTL and LB groups, with 10% of CTL pups versus 50% of LB pups exhibiting nonapproach behavior. No deficits were observed in the maternal preference test at P18 or in social exploration at P33, underscoring the robustness of these attachment-like behaviors despite severe perturbations in maternal care. Collectively, these findings reveal important similarities and differences in the effects of LB on attachment-like behavior in rats and mice and lay the groundwork for further investigation into the neurobiological underpinnings of attachment in mice.

## Synthesis

Reviewing Editor: Benjamin Deneen, Baylor College of Medicine Decisions are customarily a result of the Reviewing Editor and the peer reviewers coming together and discussing their recommendations until a consensus is reached. When revisions are invited, a fact-based synthesis statement explaining their decision and outlining what is needed to prepare a revision will be listed below. The following reviewer(s) agreed to reveal their identity: Maya Opendak. Note: If this manuscript was transferred from JNeurosci and a decision was made to accept the manuscript without peer review, a brief statement to this effect will instead be what is listed below.

The reviewers find the work to be of potential interest, but have highlighted several shortcomings that need to be addresses. In particular, it seems that the manuscript overlooks prior work in this area, and hence is lacking proper citations. This also leads to a seemingly over interpretation of the current findings. In addition to reframing the manuscript through the inclusion of these omitted references, there are several control experiments and additional experimental considerations that need to be implemented in order to proceed with this manuscript.
