# Supplementary figures and images for: Erratic Maternal Care Induces Avoidant-Like Attachment Deficits in a Mouse Model of Early Life Adversity
Source: eNeuro. 2025 Nov 6;12(11):ENEURO.0249-25.2025. doi: 10.1523/ENEURO.0249-25.2025 (PMC12614878; doi:10.1523/ENEURO.0249-25.2025)

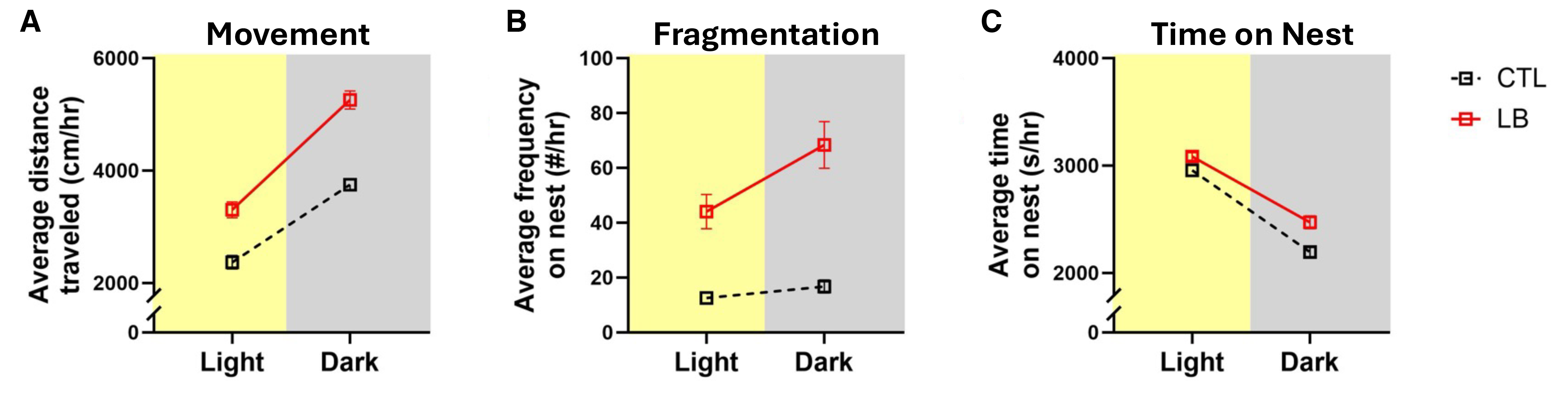

Supplement: Figure 1-1 — Differences in Maternal Behavior Are More Prominent During the Dark Phase. Maternal behavior is shown for the light and dark phases of P3-6. (A) Average hourly distance travelled. Rearing: F(1, 39) = 62.72, P < 0.0001; Light/dark: F(1, 39) = 266.8, P < 0.0001; Subject: F(39, 39) = 2.28, P = 0.0058; Rearing x Light/dark interaction: F(1, 39) = 7.96, P = 0.0075. Post-hoc analysis: CTL vs LB (light phase), CTL vs LB (dark phase), Light vs Dark (CTL), and Light vs Dark (LB) all P < 0.0001. (B) Average hourly frequency on the nest. Rearing: F(1, 39) = 34.28, P < 0.0001; Light/dark: F(1, 39) = 28.61, P < 0.0001; Subject: F(39, 39) = 7.07, P < 0.0001; Rearing x Light/dark interaction: F(1, 39) = 14.14, P = 0.0006. Post-hoc analysis: CTL vs LB (light phase) P < 0.0001, CTL vs LB (dark phase) P < 0.0001, Light vs Dark (CTL) P = 0.26, Light vs Dark (LB) P < 0.0001. (C) Average hourly time on the nest. Rearing: F(1, 39) = 14.66, P = 0.0005; Light/dark: F(1, 39) = 446.2, P < 0.0001; Subject: F(39, 39) = 2.63, P = 0.0016; Rearing x Light/dark interaction: F(1, 39) = 5.22, P = 0.028. Post-hoc analysis: CTL vs LB (light phase) P = 0.43, CTL vs LB (dark phase) P < 0.0001, Light vs Dark (CTL) P < 0.0001, Light vs Dark (LB) P < 0.0001. N = 21 litters per rearing condition. Analyzed by 2 × 2 rmANOVA with post-hoc uncorrected Fisher’s least significant difference test. Download Figure 1-1, TIF file. [file eneuro-12-ENEURO.0249-25.2025-s006.tif]

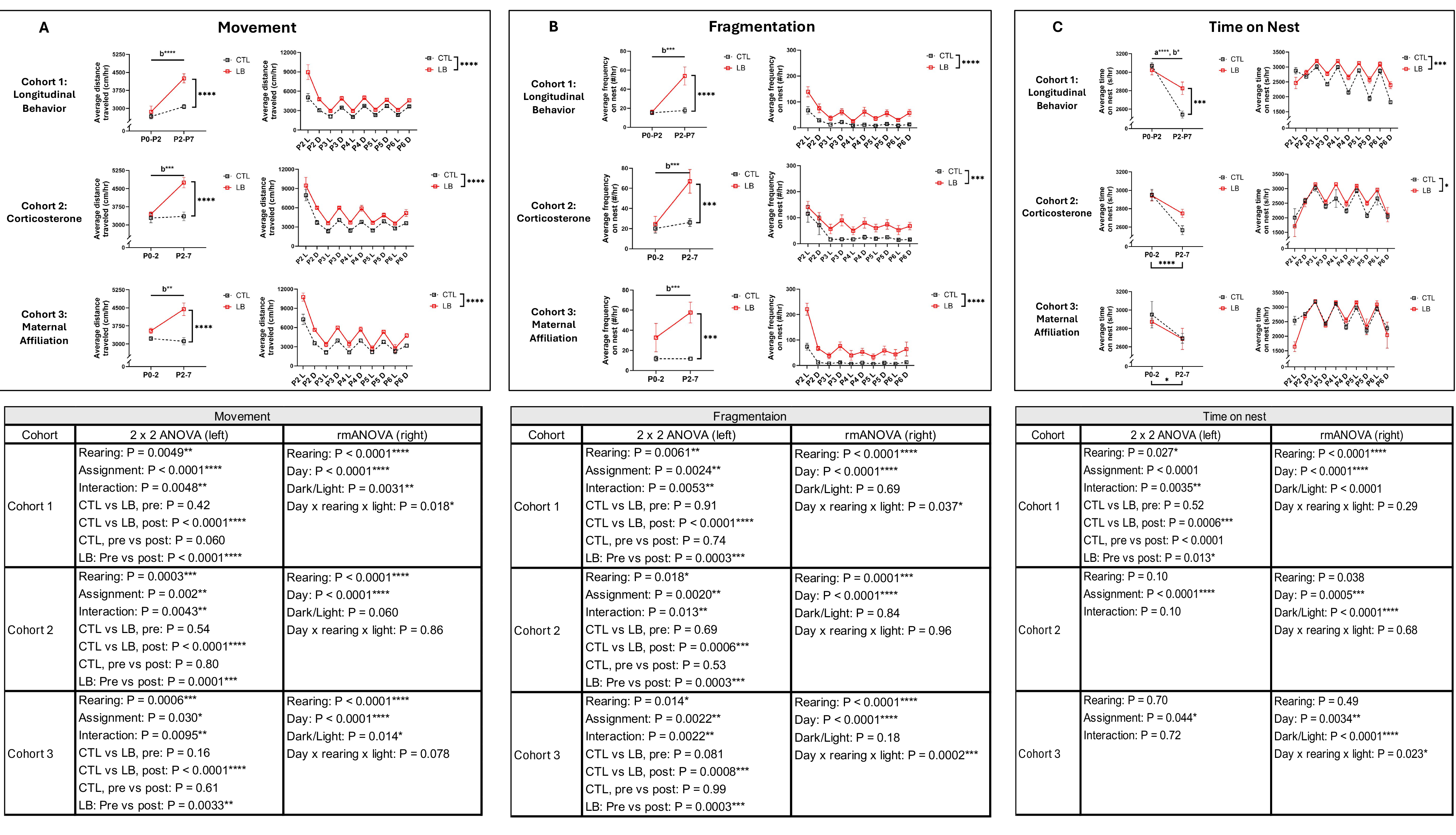

Supplement: Figure 1-2 — Reproducible and Robust Effects of Rearing Observed for Distance Traveled and Maternal Fragmentation, But Not for Time on Nest. Effects on maternal movement (A), fragmentation (B), and time on nest (C) for the three cohorts tested. Cohort 1 (n = 6 litters/condition) was used for longitudinal behavior (P8, P18, & P33), cohort 2 (n = 9 litters/condition) was used for P7 corticosterone measurements, and cohort 3 (n = 6 CTL, 5 LB litters) was used for P13 maternal affiliation behavior. Download Figure 1-2, TIF file. [file eneuro-12-ENEURO.0249-25.2025-s007.tif]

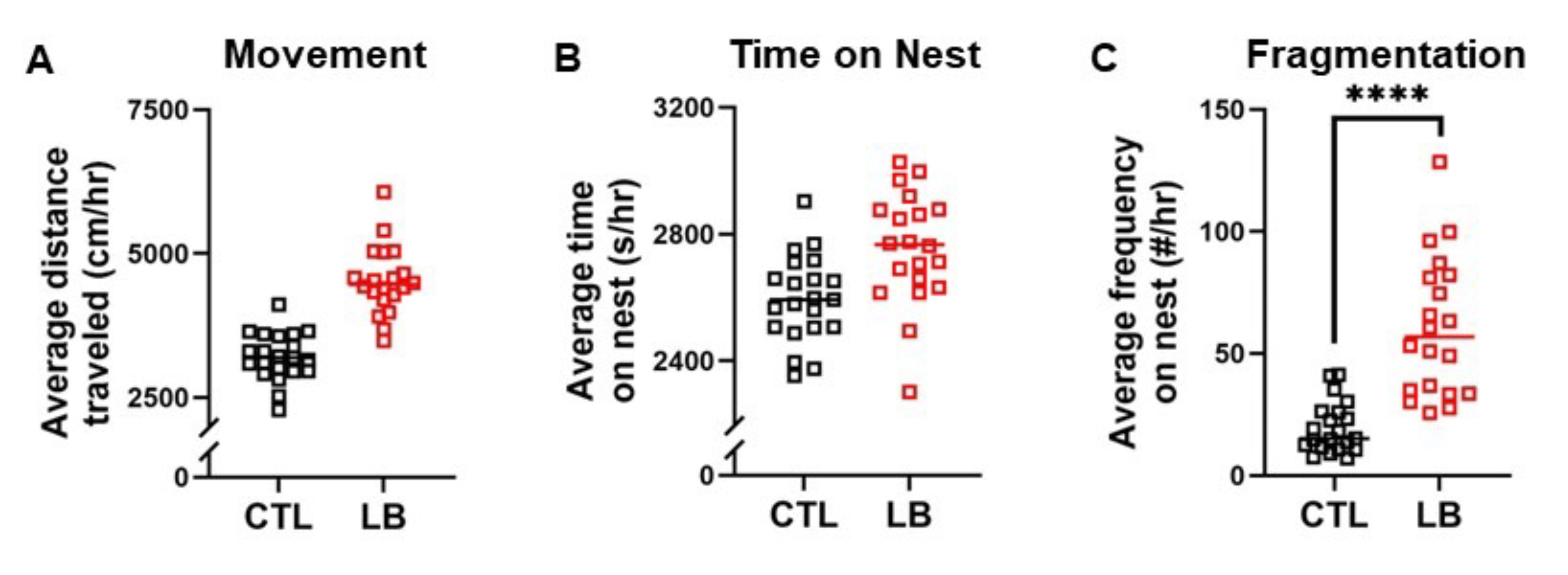

Supplement: Figure 1-3 — Effects of Rearing on Variance in Dam’s Behavior from P2 to P7. (A) Variance in movement, F (19,20) = 2.20, P = 0.13. (B) Variance in time on nest, F (19,20) = 1.69, P = 0.25. (C) Variance in fragmentation, F (19,20) = 7.47, P < 0.0001. N = 20-21 litters per rearing condition. Download Figure 1-3, TIF file. [file eneuro-12-ENEURO.0249-25.2025-s008.tif]

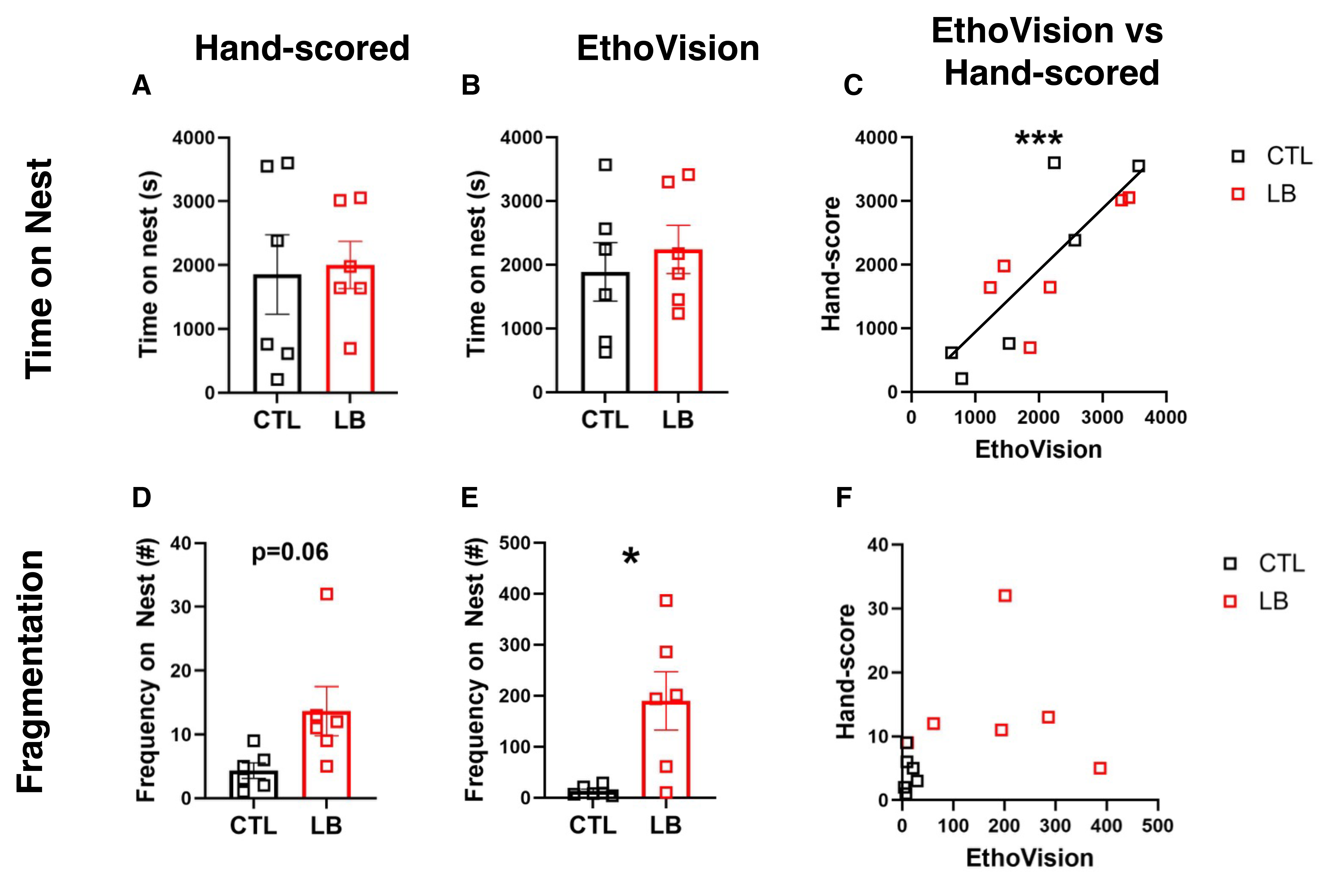

Supplement: Figure 1-4 — Comparison of Hand-Scoring and Automated EthoVision Tracking. Results from hand-scoring and automated EthoVision tracking for one hour of time on nest (A-C) and maternal fragmentation (D-F). (A) Hand-scored values for dams’ time on nest (F(5,5) = 2.84, P = 0.84). (B) EthoVision-derived time on nest values for the same period (F(5,5) = 1.48, P = 0.57). (C) Correlation between hand-scored and EthoVision values for time on nest (CTL and LB combined; r = 0.83, R² = 0.69, P = 0.0008). (D) Hand-scored values for dams’ nest entries and exits (F(5,5) = 10.23, P = 0.06). (E) EthoVision fragmentation values for the same period (F(5,5) = 203.4, P = 0.027). (F) Correlation between hand-scored and EthoVision fragmentation values (CTL and LB combined; r = 0.37, R² = 0.14, P = 0.24). N = 6 litters per condition. Welch’s t-tests were used for panels A, B, D, and E, Pearson correlations for panels C and F. Download Figure 1-4, TIF file. [file eneuro-12-ENEURO.0249-25.2025-s009.tif]

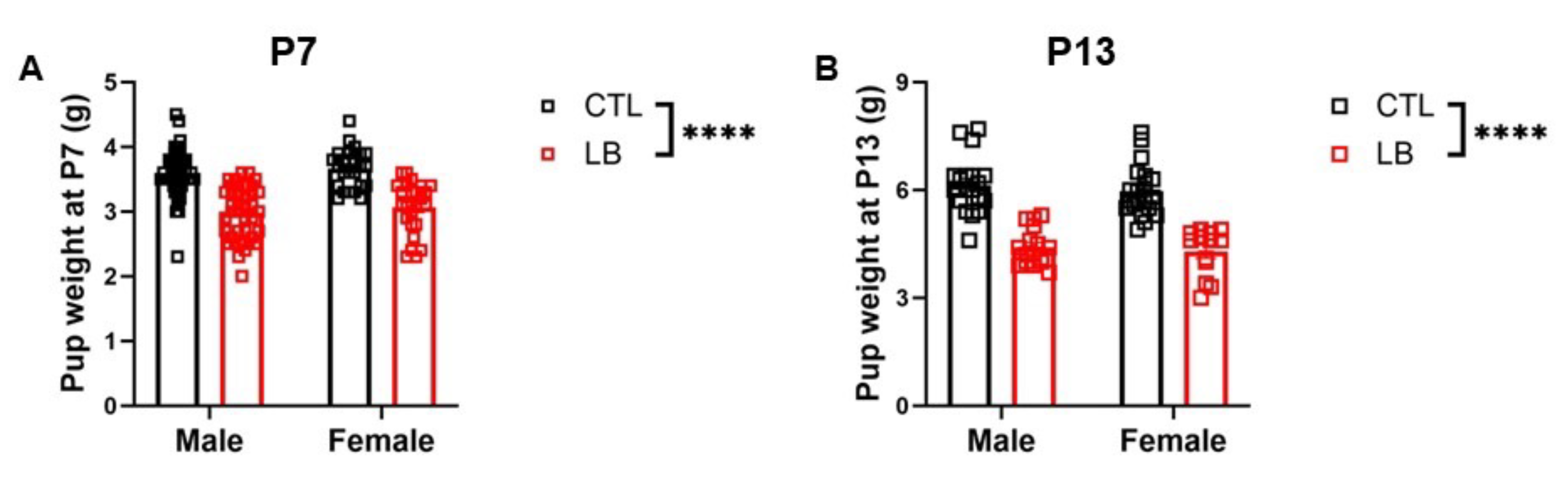

Supplement: Figure 2-1 — Pup Weights at P7 and P13. Pup weights from cohorts 2 (A) and 3 (B). (A) LB pups weigh significantly less than CTL pups at P7 without an effect of sex. N = 24-40 per group, from N = 9 litters per rearing condition. Rearing: F(1, 124) = 74.84, P < 0.0001; Sex: F(1, 124) = 1.227, P = 0.27; Interaction: F(1, 124) = 0.0046, P = 0.95. (B) LB pups weigh significantly less than CTL pups at P13 without an effect of sex. N = 13-20 per group, from N = 6 CTL and 5 LB litters. Rearing: F(1, 64) = 101.9, P < 0.0001; Sex: F(1, 64) = 0.79, P = 0.38; Interaction: F(1, 64) = 0.017, P = 0.90. Analysis by 2-way ANOVA. Download Figure 2-1, TIF file. [file eneuro-12-ENEURO.0249-25.2025-s010.tif]

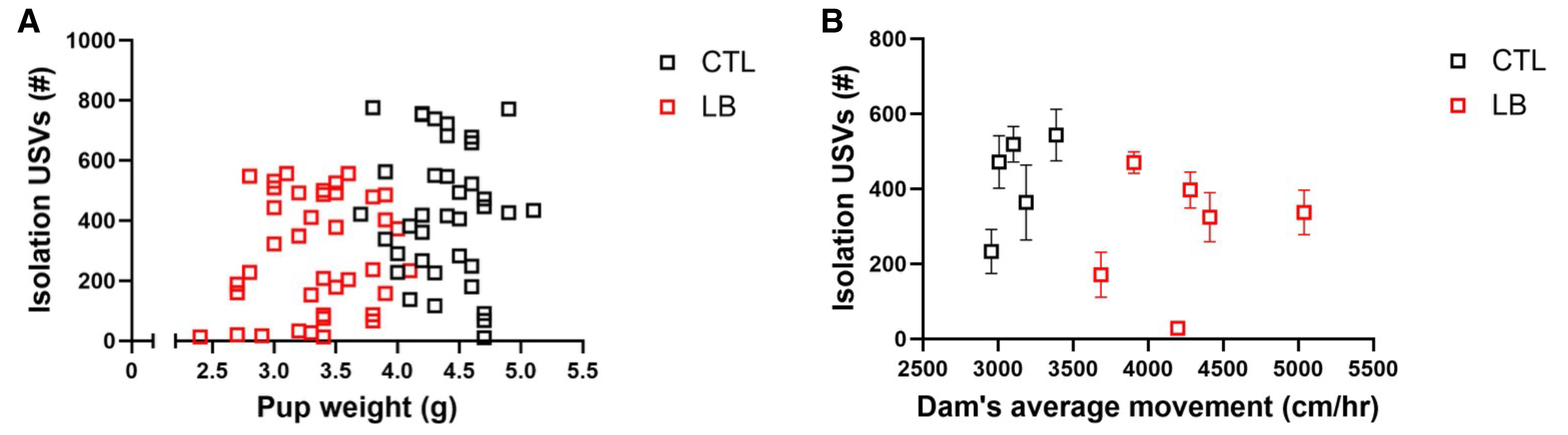

Supplement: Figure 4-1 — P8 Isolation USVs Do Not Correlate to Pup Weight or Dam Behavior. (A) Correlation between P8 CTL and LB pup weights and number of USVs during the isolation phase of the maternal buffering test. N = 37-39 per condition, sexes are combined for analysis since neither behavior nor weight varied by sex. CTL: R2 = 0.0053, P = 0.67; LB: R2 = 0.017, P = 0.43. (B) Dams’ average movement P2-7 does not correlate with pups’ USVs during isolation at P8. CTL: R2 = 0.36, P = 0.29; LB: R2 = 0.03, P = 0.74. Download Figure 4-1, TIF file. [file eneuro-12-ENEURO.0249-25.2025-s011.tif]

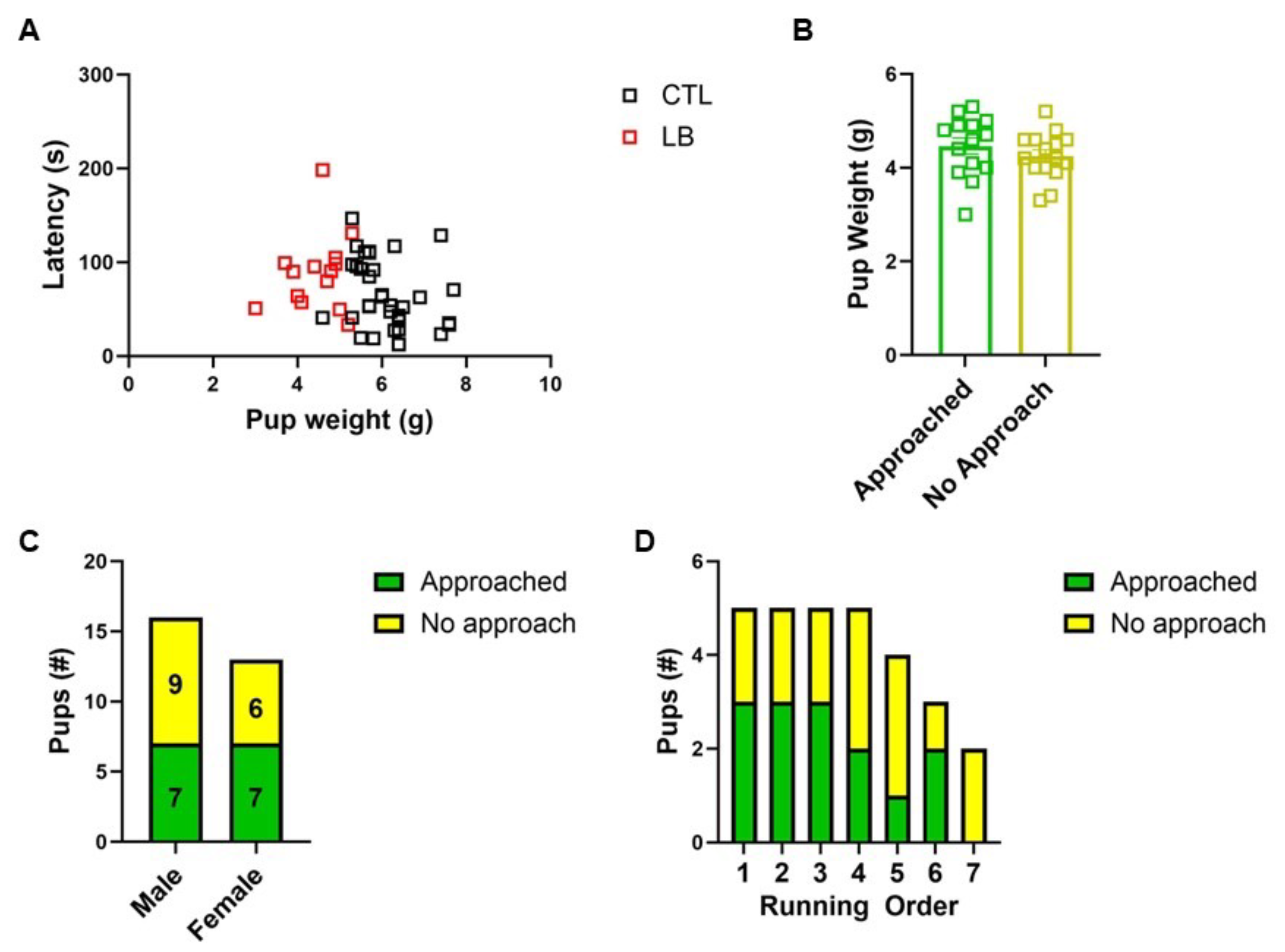

Supplement: Figure 5-1 — Effects of Body Weight, Sex, and Order of Testing on Approach behavior in P13 LB pups. (A) Correlation between pup body weight and latency to reach the dam for CTL and LB pups that approached. CTL: R2 = 0.075, P = 0.11; LB: R2 = 0.037, P = 0.51. (B) Body weight, t(27) = 0.98, P = 0.34. (C) Sex, Fisher’s exact test, P = 0.72. (D) Testing order. Fisher’s exact test, P = 0.83. N = 29 pups from N = 5 litters, sexes combined for A & C. Download Figure 5-1, TIF file. [file eneuro-12-ENEURO.0249-25.2025-s012.tif]

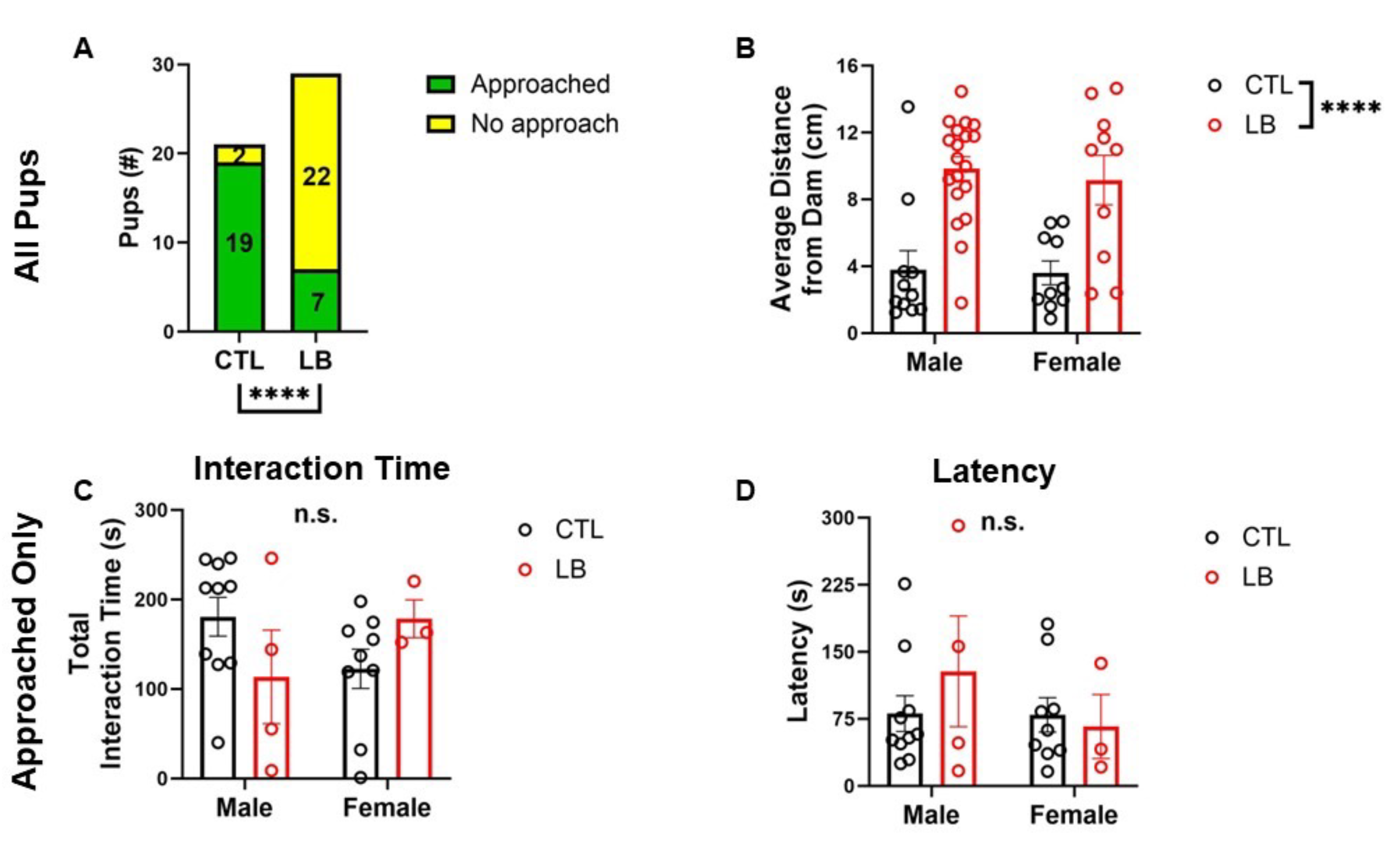

Supplement: Figure 5-2 — Similar P13 Results in a Cohort of Standard-housed Pups. (A) 19 out of 21 (90%) of CTL pups approached the dam vs. 7 out of 29 (24%) of LB pups: Fisher’s exact test P < 0.0001. (B) Average distance from the dam. Rearing: F (1, 46) = 32.42, P < 0.0001; Sex: NS; Interaction: NS. (C) Total interaction time among pups that approached the dam. Rearing, sex and interaction were all NS. (D) Latency to approach the dam among pups that did so. Rearing, sex and interaction were all NS. 2-way ANOVA in B, C, and D. N = 10-19 per sex and rearing condition (total of 21 CTL and 29 LB pups), from N = 3 CTL and 4 LB litters. NS-non-significant, P > 0.05. Download Figure 5-2, TIF file. [file eneuro-12-ENEURO.0249-25.2025-s013.tif]

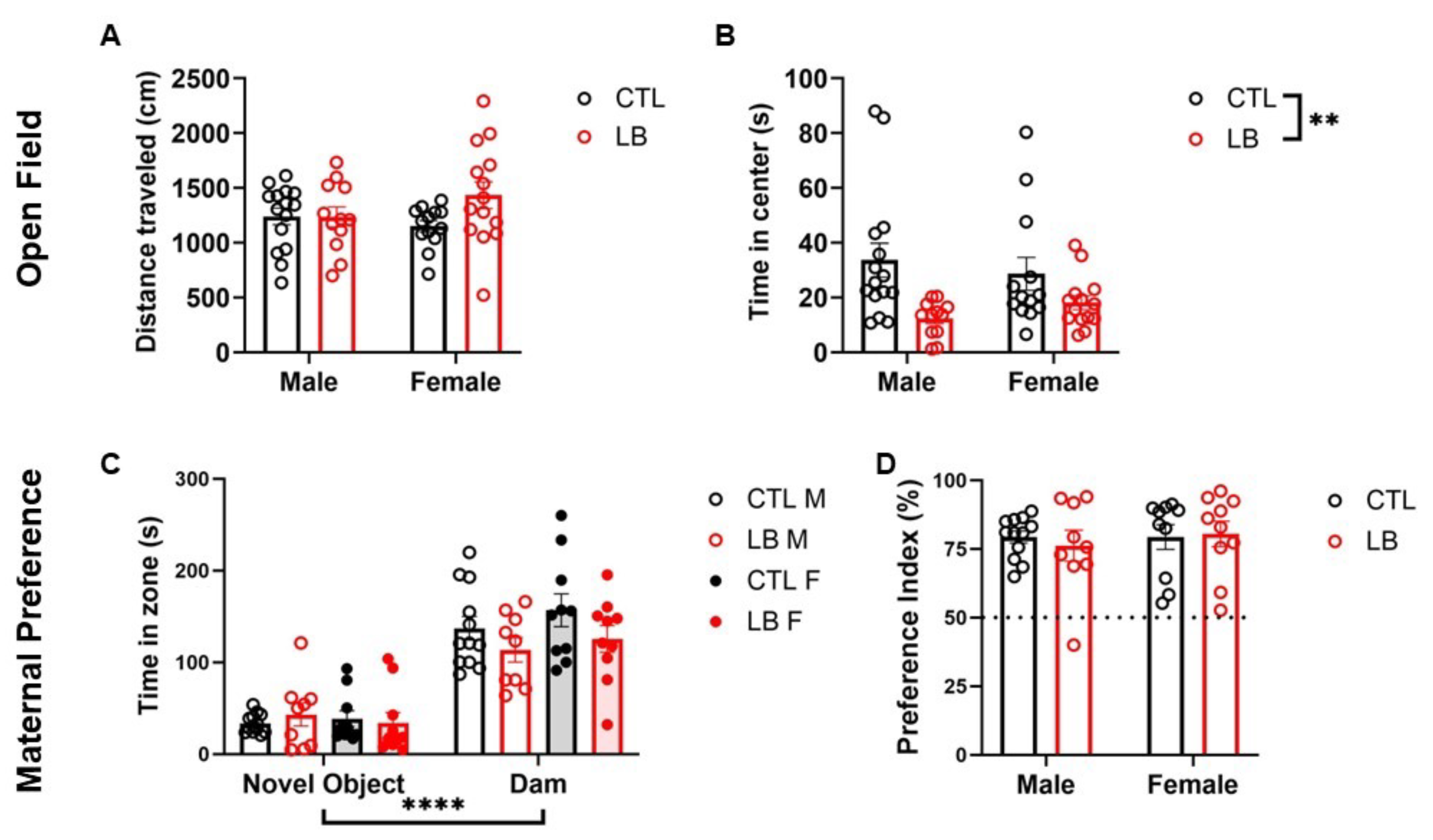

Supplement: Figure 6-1 — Similar P18 Results in a Cohort of Standard-housed Pups. (A) Distance moved during the open field test. Rearing: F (1, 50) = 2.39,P = 0.13; Sex: F (1, 50) = 0.37, P = 0.55; Interaction: F (1, 50) = 2.56, P = 0.12. (B) Time spent in the center of the arena. Rearing: F (1, 50) = 11.10, P = 0.0016; Sex: F (1, 50) = 0.0076, P = 0.93; Interaction: F (1, 50) = 1.25, P = 0.27. (C) Time spent near the novel object and the dam. Zone: F (1, 37) = 116.5, P < 0.0001; Rearing: F (1, 37) = 2.19, P = 0.15; Sex: F(1, 37) = 0.65, P = 0.42; Zone x Sex: F(1, 37) = 0.1, P = 0. 32; Zone x Rearing: F (1, 37) = 2.81, P = 0.10; Sex x Rearing: F (1, 37) = 0.41, P = 0.52; Zone x Sex x Rearing: F (1, 37) = 0.033, P = 0.86. (D) Preference index. Rearing: F (1, 37) = 0.057, P = 0.81; Sex: F (1, 37) = 0.26, P = 0.62; Interaction: F (1, 37) = 0.26, P = 0.61. N = 9-12 pups per group from N = 4-5 litters. 2 × 2 ANOVA in A, B, & D. 3-way rmANOVA in C. Download Figure 6-1, TIF file. [file eneuro-12-ENEURO.0249-25.2025-s014.tif]
